# Comparison of diagnostic methods and analysis of socio-demographic factors associated with *Trichomonas vaginalis* infection in Sri Lanka

**DOI:** 10.1371/journal.pone.0258556

**Published:** 2021-10-13

**Authors:** Sayuri Herath, Thivya Balendran, Akila Herath, Devika Iddawela, Susiji Wickramasinghe

**Affiliations:** 1 Department of Medical Laboratory Sciences, Faculty of Health Sciences, The Open University of Sri Lanka, Nugegoda, Sri Lanka; 2 Department of Parasitology, Faculty of Medicine, University of Peradeniya, Peradeniya, Sri Lanka; 3 Department of Statistics and Computer Science, Faculty of Science, University of Peradeniya, Peradeniya, Sri Lanka; Babol University of Medical Science, ISLAMIC REPUBLIC OF IRAN

## Abstract

**Background:**

*Trichomonas vaginalis* infection is underreported due to nonspecific clinical presentation and the nonavailability of sensitive laboratory diagnostic tests at the clinical setup. Hence, this study was designed to compare the sensitivity and specificity of microscopy and culture methods with polymerase chain reaction (PCR). The socio-demographic factors associated with the infection were explored.

**Methods:**

The study was carried out at the National Sexually Transmitted Diseases and Acquired Immuno Deficiency Syndrome Control Programme in Colombo and Sexually Transmitted Diseases and Acquired Immuno Deficiency Syndrome Control Programme in Kandy. Samples were collected from a total of 385 patients including, 272 females (70.7%) and 113 males (29.3%), and tested using microscopy (wet mount and Giemsa staining), culture, and PCR. Genus-specific primer set (TFR1/TFR2) that amplifies 5.8S rRNA and species-specific primer sets (TV16Sf-2/TV16Sr-2 and TVK3/7) that amplifies 18S rRNA and repetitive DNA, respectively, were used. Patient’s socio-demographic and sexual behaviour data were obtained using a standard interviewer-administered questionnaire. Data were analyzed with R statistical software Version 3.6.3.

**Results:**

The overall prevalence of trichomoniasis was 4.4% (17/385). Of these, six (1.6%) were positive for microscopic examination, 7 (1.8%) were positive for culture, and 13 (3.4%) for TVK3/7, 15 (3.9%) for TV16Sf/r, and TFR1/2 17 (4.4%) were positive for PCR. Sensitivities of PCR using TFR1/2, TV16Sf/r, and TVK3/7 primer sets were 100%, 88.20%, and 76.50%, respectively, against the expanded gold standard. Trichomoniasis was associated with age above 36 (*p* = 0.033), not using condoms in last three months (*p* = 0.016), multiple sex partners (*p* = 0.001), reason for attendance (*p =* 0.027), symptomatic nature (*p* = 0.015), and the presence of other sexually transmitted diseases (*p* = 0.001).

**Conclusions:**

The study highlighted that age over 36 years, multiple sex partners, not using condoms, reason for attendance, symptomatic nature, and having other sexually transmitted diseases can increase the risk of acquiring trichomoniasis. Furthermore, this study confirmed PCR as highly sensitive and specific diagnostic test for the diagnosis of trichomoniasis in comparison to microscopy and culture methods.

## Introduction

*Trichomonas vaginalis* is a highly motile, aerotolerant, eukaryotic parasite that resides in the urogenital tract of humans [1]. It causes trichomoniasis, the most common, non-viral sexually transmitted infection in humans [1, 2]. The center for disease control and prevention (CDC) has listed it as one of the neglected parasitic infections and recommends special measures to contain the disease [3, 4]. The world health organization (WHO) has estimated 187.1 million cases of *T*. *vaginalis* infection annually around the globe [5]. Incidence is varied among studied populations. There are 28.7 million cases of *Trichomonas* infection in South East Asia [6].

Infected females are usually symptomatic [7] and commonly show purulent and foul-smelling vaginal discharge, vulval irritation, itching, dysuria, abdominal pain, and dyspareunia [8–10]. Furthermore, it causes punctuate hemorrhagic lesions, which appear as strawberry cervix [11]. However, these typical clinical symptoms are seen only in 20% of trichomoniasis cases [12]. Infection with the organism is generally asymptomatic in the majority of men [9, 11].

The main public health importance of the disease is the high incidence of premature destruction of membranes, preterm birth, low birth weight neonates, stillbirth, and neonatal death in infected pregnant women [9, 13–15]. In addition, it can cause infertility, endometritis, pelvic inflammatory disease, and cervical neoplasia [9, 15]. Furthermore, *Trichomonas* infection can enhance the susceptibility of patients to human immunodeficiency virus (HIV) infection. Diagnosis of trichomoniasis based on the clinical features is not sufficient due to the nonspecific nature of the clinical presentation, which overlaps with the clinical features of other sexually transmitted diseases [8, 16]. Hence, the etiological diagnosis by laboratory investigations is essential for proper diagnosis and management of the disease.

The most commonly performed laboratory diagnostic method is a wet mount [11, 17]. Direct observation of the pear-shaped trichomonads with their characteristic jerky or tumbling motility is considered 100% specific for the organism. However, the negative results cannot exclude trichomoniasis because of its low sensitivity (38%-82%) [18]. Other than the wet mount, Giemsa staining [19], modified Field’s staining [2], and liquid-based Pap tests [8] are used to demonstrate *T*. *vaginalis* in vaginal smears. The disadvantages of staining methods include loss of motility of trophozoites due to the fixation. Moreover, *Trichomonas* does not always bear the typical pear shape, which leads to difficulty in identification [11]. Culture in microaerophilic conditions is calculated to be 70–80% sensitive [20]. However, a density of 10^2^ trichomonads per milliliter of sample is needed for a culture to be rendered positive [17]. But the culture is considered a gold standard for the diagnosis of *T*. *vaginalis* infection [8, 20].

Numerous rapid diagnostic kits are available, whereas their performance characteristics are not yet published and problematic [21]. Fluorescent antibody test, enzyme-linked immunosorbent assay, loop-mediated isothermal amplification (LAMP) assay, and hybridization test are also used to detect *T*. *vaginalis* infection [22]. Polymerase chain reaction (PCR) is more sensitive than culture and other techniques in detecting *T*. *vaginalis* [23, 24]. PCR is performed from a variety of samples, including urine and vaginal swabs [25]. Unlike other available techniques, one organism per PCR mixture is adequate to give a positive result [25].

There is a lack of information regarding the prevalence, risk factors, and diagnostic methods of trichomoniasis in Sri Lanka. Herath *et al*. (2012) [26] reported a 7.2% prevalence in Colombo city. A recent study done by Samarawickrema *et al*. (2015) [27] reported a 2.3% prevalence of trichomoniasis in Sri Lanka. However, the National Sexually Transmitted Diseases and Acquired Immuno Deficiency Syndrome Control Programme (NSACP) shows a long-term decreasing trend of the disease over the past years, and it accounts for only a 0.32 rate per 100000 adults in 2018 [28]. However, trichomoniasis has been made a notifiable disease in Sri Lanka as it mostly affects women of reproductive age. Hence, the use of a more sensitive test to diagnose trichomoniasis in clinical settings is important as it affects the epidemiology and clinical management of trichomoniasis in Sri Lanka. Therefore, this study was designed to establish a PCR-based diagnostic test for diagnosis of trichomoniasis in the Sri Lankan clinical settings and to compare the sensitivity and specificity of commonly used microscopy (wet mount and Giemsa staining) and culture method with PCR. The socio-demographic factors and sexual behaviors associated with *Trichomonas* infection in Sri Lanka were also investigated.

## Materials and methods

### Study design and population

A descriptive cross-sectional study was carried out in NSACP in Colombo and Sexually Transmitted Diseases and Acquired Immuno Deficiency Syndrome (STD/AIDS) Control Programme in Kandy from April 2017 to December 2019. The minimum sample size (n = 310) was calculated using 2.3% prevalence [27] with a 95% confidence interval [29] (S1 Appendix). However, to increase the power of the study, 385 samples were collected during the study period.

Three hundred and eighty-five patients were screened irrespective of the presence or absence of the symptoms. Patients aged between 15–55 years who had undergone vaginal/urethral examination were included in the study. Female patients who were menstruating or having any vaginal bleeding at the time of vaginal examination and those who had metronidazole and or tinidazole within one week before sampling were excluded [1].

### Ethical statement

Ethical clearance for this study was obtained from the Ethics Review Committee, Faculty of Medicine, University of Peradeniya, Sri Lanka (Ethical approval No:2016/EC/92). Participants were informed about the research and procedures and written informed consent was obtained for sample and data collection (S2 Appendix). For participants aged less than 18, consent was obtained from parents or guardians.

### Data collection and sample processing

For the study, patients’ demographic data (age, gender, occupation, and level of education), clinical history (genitourinary symptoms, duration of symptoms, signs, medication, miscarriages/stillbirth, previous STD or AIDS, and results of previous HIV tests), sexual behaviour (types of partner, number of partners, sexual orientation, and condom usage), and details regarding drug or alcohol consumption were obtained with the standard interviewer-administered questionnaire by a clinician (S1 Appendix).

Each patient was subjected to speculum-assisted genital examination by a clinician, as a part of the routine clinical examination process carried out at the NSACP, Colombo, and STD/AIDS Control Programme, Kandy. Four vaginal swabs were collected from the posterior fornix of each female patient using commercially available 10 μl sterile, plastic, disposable inoculating loops. One swab from the posterior fornix was inoculated into a 1.5 ml microcentrifuge tube with 0.5 ml of 0.9% sterile normal saline and mixed well for the wet mount. The second swab was rolled on the slide thoroughly, and it was used for Giemsa staining. The third swab was inoculated into a 5 ml sterile screw cap bottle with a commercially available *Trichomonas* culture medium (Oxoid 2) for culture. The final swab was used for genomic DNA extraction. It was inoculated into a 1.5 ml microcentrifuge tube with 0.5 ml of 0.9% sterile normal saline and mixed thoroughly. The urine samples were collected from male patients into 15 ml sterile plastic conical centrifuge tubes, and centrifuged deposit was used for microscopy (wet mount and Giemsa staining), culture, and genomic DNA extraction. Double-blinded laboratory diagnosis was performed, where clinical history of the participants was not revealed to the microscopist and vice versa.

### Wet mount preparation

A drop of normal saline (0.9%) mixed with a fresh vaginal swab was placed on a clean microscopic slide to prepare a wet mount. A drop of centrifuged urine sediment was used for male patients. Wet mounts were examined under high power (x40) to detect motile pear-shaped trophozoites. The presence of pear-shaped trophozoites with characteristic jerky or tumbling motility was considered as a positive.

### Giemsa staining

The smear prepared on the glass slide was air-dried and fixed with methanol for 1 minute. Then, the smear was stained using Giemsa. Subsequently, the slide was washed using clean tap water and placed vertically to drain and dry the smear. Stained smears were then observed under (x100) magnification systematically to cover the whole smear. The presence of violet, pear-shaped trophozoites with four free flagella was considered as a positive [30].

### In vitro culture

After transporting into the laboratory, the cap of the inoculated culture bottles was loosened and incubated at 37°C in a 5% CO_2_ incubator for three to five days. The cultures were examined daily microscopically by placing the drop of culture on a glass slide. The presence of pear-shaped trophozoite with characteristic jerky or tumbling motility was considered as a positive.

### DNA extraction and polymerase chain reaction (PCR)

Genomic DNA was extracted from 385 samples using Pure Link^TM^ Genomic DNA Mini extraction kit (Invitrogen, Life Technologies, USA) according to the manufacturer’s protocol. All 385 samples were subjected to PCR using three different sets of primers (Table 1).

**Table 1:** Amplified regions and the genus-specific and the species-specific primers used in the study.

Primer number	Amplified region and size of the amplicon	Primer name and the sequence (5’-3’)	Reference
Primer set 1 (Genus specific)	5.8S rRNA	F: TFR1 (TGCTTCAGTTCAGCGGGTCTTCC)	[17]
	(350–400 bp)	R: TFR2 (CGGTAGGTGAACCTGCCGTTGG)	
Primer set 2 (Species specific)	18S rRNA	F: TV16Sf-2 for (TGAATCAACACGGGGAAAC)	[17]
	(323 bp)	R: TV16Sr -2 (ACCCTCTAAGGCTCGCAGT)	
Primer set 3 (Species specific)	Repetitive DNA	F: TVK3 (ATTGTCGAACATTGGTCTTACCCTC)	[23]
	(260–350 bp)	R: TVK7 (TCTGTGCCGTCTTCAAGTATGC)	

F: Forward, R: Reverse.

PCR amplifications were carried out in an automated thermocycler (Amplitronix^TM^ 6 version 1.0.4, USA). PCR reaction mixture had 2.5 μl 10x reaction buffer, 2 μl of 2.5 mM deoxynucleotide triphosphate (dNTPs), 2 μl of 50 mM MgCl_2,_ 0.25 μl of 5U/μl Taq DNA polymerase, 1.5 μl 10 pmol forward and reverse primers, 5 μl template DNA, a total volume of 25 μl. PCR conditions for all three primer sets were as follows; initial denaturation at 94°C for 3 min, 35 cycle denaturation at 94°C for 30 s, annealing at 60°C for 30 s, extension at 72°C for 1 min, final extension at 72°C for 7 min. Positive and negative controls were used for each PCR reaction.

### Calculation of expanded gold standard

The sensitivity, specificity, positive predictive value (PPV), and negative predictive value (NPV) of diagnostic tests were calculated in comparison to the expanded gold standard. Samples that were tested positive for the genus-specific primer set and one of the two species-specific primer sets (TV16Sf-2/TV16Sr-2 or TVK3/TVK7) were considered as an expanded gold standard for *T*. *vaginalis* infection [31] (S3 Appendix).

### Statistical analysis

Statistical analysis was done using R statistical software (Version 3.6.3). Positive or negative status for trichomoniasis was considered as the dependent variable, while demographic factors, clinical variables, and sexual behaviours were considered as independent variables. The association of the categorical variables was evaluated by Pearson’s chi-square test and its non-parametric alternative Fisher’s exact test. The probability value *p*<0.05 was considered statistically significant. Odds Ratio (OR) was used to find the direction and magnitude of the association of the categorical variables. Correlations between the positive or negative status for *Trichomonas* infection and other independent variables were calculated by the multiple logistic regression.

## Results

A total of 385 participants (272 females, 70.7% and 113 males, 29.3%) were included in the study. The prevalence of *T*. *vaginalis* estimated in this study was 1.6% (6/385) by microscopy and 1.8% (7/385) by culture. Six samples were positive for both Giemsa staining and wet mount (Table 2, S1 Fig). Six microscopy-positive samples and additional one microscopy-negative sample were positive for culture.

**Table 2:** Prevalence of *T*. *vaginalis* infection by microscopy, culture, and PCR.

Type of diagnostic tests	Number of positive samples	Percentage (%)
Microscopy		
Wet mount	6	1.6
Giemsa staining	6	1.6
Culture	7	1.8
PCR	17	4.4

* Same six samples were positive for both Giemsa staining and wet mount. Seven culture-positive samples contain all six microscopy-positive samples. Seventeen PCR-positive samples include all seven culture-positive samples.

### PCR

**Fig 1:**
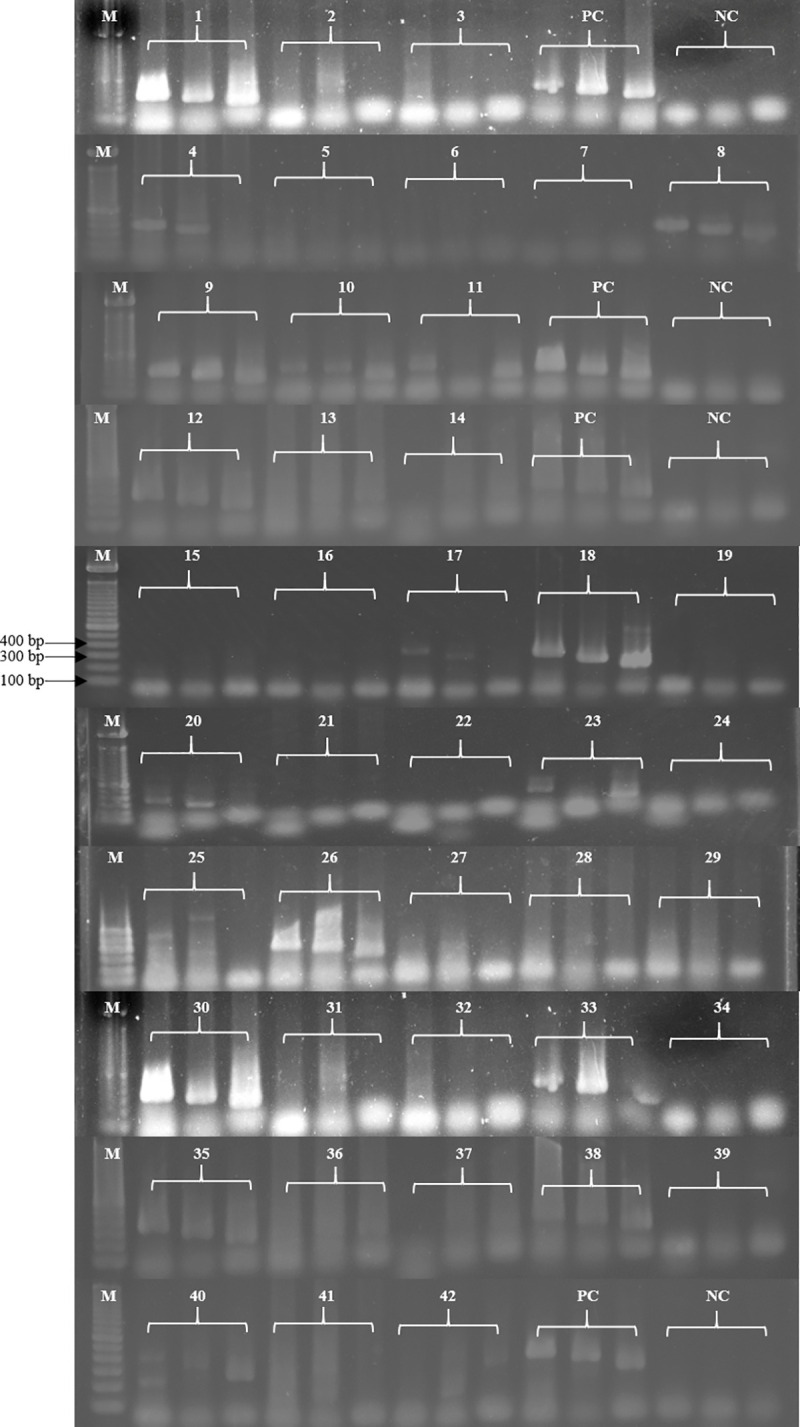
Diamond™ nucleic acid dye stained agarose gel image of PCR amplified products of 5.8S rRNA, 18S rRNA, and repetitive DNA regions for all three primers TFR1/2, TV16Sf/r, and TVK3/7. M: 100 bp molecular marker; PC: positive control; NC: negative control. Among the samples, sample number 1, 8, 9, 10, 12, 18, 26, 30, 35, 38, and 40 showed positivity for all three primer sets TFR1/2, TV16Sf/r, and TVK3/7; sample number 4, 7, 20, and 33 showed positivity only for TFR1/2 and TV16Sf/r; and sample number 11 and 23 showed positivity only for TFR1/2 and TVK3/7.

Of 385 samples, 17 samples were positive for the genus-specific PCR. Of these, 11 samples were positive for both species-specific primer sets (TV16Sf/r and TVK3/7). Four samples were positive only for TV16Sf/r primer set (Fig 1), and 2 samples were positive for TVK3/7 primer set (Table 3). Seven culture-positive samples and additional ten culture-negative samples were positive for PCR. Accordingly, the overall prevalence of *T*. *vaginalis* in the study population was 4.4% (17 out of 385 participants).

**Table 3:** Positivity using three different primer sets in PCR.

Number of Samples		PCR	
	TFR1/2	TV16Sf/r	TVK3/7
11	**+**	**+**	**+**
4	+	+	-
2	+	-	+
**Total Number of positive samples**	**17**	**15**	**13**

* “+”: positive, “-”: negative.

### Sensitivity and specificity of each diagnostic test

Comparison of each diagnostic test with expanded gold standard was carried out, and the sensitivity, specificity, PPV, and NPV of each diagnostic test were calculated (S3 Appendix) and tabulated in Table 4. Accordingly, the highest sensitivity was estimated for PCR (100.00%) followed by culture (41.20%) and microscopy (35.30%), respectively (Table 4). 100% specificity was obtained for all the diagnostic tests.

**Table 4:** Performance of each diagnostic test compared to the expandard gold standard.

Diagnostic test	Sensitivity (%)	95% CI	Specificity (%)	95% CI	AUC	PPV (%)	NPV (%)
**Microscopy method**							
Wet mount	35.30	15.26 to 61.38	100.00	98.71 to 100.00	0.6	100.00	97.10
Giemsa staining	35.30	15.26 to 61.38	100.00	98.71 to 100.00	0.6	100.00	97.10
**Culture**	41.20	19.43 to 66.55	100.00	98.71 to 100.00	0.6	100.00	97.35
**PCR**							
Primer set TFR1/2	100.00	77.08 to 100.00	100.00	98.71 to 100.00	-	100.00	100.00
Primer set TV16Sf/r	88.20	62.25 to 97.94	100.00	98.71 to 100.00	0.9	100.00	99.46
Primer set TVK3/7	76.50	76.50 to 92.18	100.00	98.71 to 100.00	0.8	100.00	98.92

CI: confidence interval, AUC: area under the curve; PPV: positive predictive value, NPV: negative predictive value.

### Socio-demography of the study population

Out of 385, the majority were females (272, 70.6%). A large proportion (163, 42.3%) of the study population were aged between 26 to 35 years (Table 5). The majority of clinic attendees (161, 41.8%) were employed, and 111 (28.8%) were unemployed. In the total population, 100 (26%) participants were commercial sex workers. Only 191 patients were symptomatic, and others were asymptomatic at the time of attending the STD clinic. Among the symptomatic patients, the majority presented with vaginal discharge with odor (60/191, 31.4%), followed by genital ulcers (28/191, 14.7%) and genital warts (17/191, 8.9%). In addition to these, 23 patients showed multiple symptoms. The majority of the study population (217/385, 56.4%) had a single sexual partner (their marital partner), whereas others had non-regular sexual partners and commercial sexual partners (Table 5).

**Table 5:** Demographic characteristics and sexual behavior of patients and the association and prevalence of trichomoniasis.

Variable		Frequency			*T*. *vaginalis* infection	*p*-value of Univariate analysis
		Female (272)	Male (113)	Total (385)		
**Demographic characteristics**						
**Civil Status**	*Single*	23	47	70	-	0.06167
	*Married*	191	62	253	13/17 (76.5%)	
	*Living together*	10	2	12	-	
	*Divorced*	37	1	38	2/17 (11.8%)	
	*Widowed*	11	1	12	2/17 (11.8%)	
**Age**	*15–25*	60	21	81	1/17 (5.9%)	0.03317
	*26–35*	109	54	163	4/17 (23.5%)	
	*36–45*	58	24	82	8/17 (47.1%)	
	*46–55*	45	14	59	4/17 (23.5%)	
**Level of education**	*1–5 grade*	14	2	16	1/17 (5.9%)	0.28250
	*6–10 grade*	85	12	97	4/17 (23.5%)	
	*G*.*C*.*E*. *O/L*	105	63	168	9/17 (52.9%)	
	*G*.*C*.*E*. *A/L*	48	31	79	1/17 (5.9%)	
	*Diploma/Degree*	11	5	16	1/17 (5.9%)	
	*No schooling*	9	0	9	1/17 (5.9%)	
**Employment status**	*Employed*	65	96	161	3/17 (17.6%)	0.13440
	*Unemployed*	107	17	124	7/17 (41.2%)	
	* *CSW*	100	0	100	7/17 (41.2%)	
**Reason for attendance**	*Voluntary*	129	92	221	7/17 (41.2%)	0.02713
	*Ref*. *OPD*	33	5	38	3/17 (17.6%)	
	*Ref*. *Courts*	59	5	64	3/17 (17.6%)	
	*Ref*. *Ward*	9	3	12	1/17 (5.9%)	
	*Ref*. *GP*	6	1	7	1/17 (5.9%)	
	*Ref*, *Blood bank*	10	1	11	1/17 (5.9%)	
	*Contacts*	16	2	18	-	
	*Clinic follow up*	0	1	1	-	
	*Medico legal*	5	0	5	-	
	*Others*	5	3	8	1/17 (5.9%)	
**Symptomatic nature**	*Symptomatic*	158	33	191		0.01474
	*Genital discharge with odor*	43	17	60	6/17 (35.3%)	
	*Genital ulcers*	18	10	28	-	
	*Genital warts*	06	11	17	-	
	*Dysuria*	15	28	43	-	
	*Pelvic pain*	13	07	20	-	
	*Multiple symptoms*	17	06	23	-	
	*Asymptomatic*	114	80	194	-	
**Sexual behavior**						
**Sexual contact**	*None*	30	15	45	-	0.83900
	*Sri Lankan*	221	91	312	15/17 (88.2%)	
	*Foreign*	13	1	14	2/17 (11.8%)	
	*Sri Lankan & Foreign*	8	6	14	-	
**Sexual orientation**	*Heterosexual*	253	63	316	16/17 (94.1%)	0.79340
	*Bisexual*	8	19	27	-	
	*Homosexual*	11	31	42	1/17 (5.9%)	
**No. of sexual partners**	*One*	166	51	217	9/17 (52.9%)	0.00183
	*Two*	13	26	39	-	
	*Three*	7	3	10	4/17 (23.5%)	
	*Four*	8	1	9	-	
	*Five or more*	56	2	58	4/17 (23.5%)	
	*None*	22	30	52	-	
**Condom use at last sex**	*No*	190	86	276	9/17 (52.9%)	0.03999
	*Yes*	67	19	86	8/17 (47.1%)	
	*None*	15	7	22	-	
**Condom use at last 3 months**	*Always*	49	12	61	1/17 (5.9%)	0.01639
	*Never*	160	61	221	9/17 (52.9%)	
	*Sometimes*	35	18	53	7/17 (41.2%)	
	*Not applicable*	28	21	49	-	
**Sextually transmitted disease**	*No STD related illness*	66	73	139	-	0.00140
	*Bacterial vaginosis*	31	0	31	7/17 (41.2%)	
	*HSV infection*	16	5	21	-	
	*Syphilis*	12	3	15	2/17 (11.8%)	
	*Non gonococcal urethritis*	33	10	43	4/17 (23.5%)	
	*AIDS*	0	0	0	-	
	*Pelvic inflammatory diseases*	4	0	4	1/17 (5.9%)	
	*Candidiasis*	20	12	32	3/17 (17.6%)	
	*Other*	0	2	2	-	

* *p*-value-probability value; CSW-Commercial sex worker; G.C.E O/L-general certificate of education ordinary level; G.C.E. A/L- general certificate of education advanced level.

### Factors associated with *Trichomonas* infection

In the present study, the majority of the infected patients were females (15/17, 88.2%), however, this was not statistically significant (*p* = 0.188). Most of the *Trichomonas* positive patients were aged between 36 and 45 years (8/17, 47.1%). On univariate analysis, age was significantly associated with *Trichomonas* infection (*p* = 0.033, OR, 0.22; 95% CI, 0.06–0.70). Multiple logistic regression showed patients older than 36 years had a greater risk of contracting the disease (OR, 1.101; 95% CI, 0650–1.699; *p* = 0.003) (Table 6). Marital status was not significantly associated with the disease occurrence (*p* = 0.062). However, the majority (13/17, 76.5%) of infected patients were married (Table 5). Half of the *T*. *vaginalis* positive individuals (9/17, 52.9%) were educated up to general certificate of ordinary level (G.C.E O/L) though the difference was not statistically significant (*p* = 0.282). This study showed that employment has no significant bearing on disease occurrence (*p* = 0.134). The equal proportion of tested positives (7/17, 41.2%) were unemployed as well as commercial sex workers (7/17, 41.2%), while only 3 (17.6%) were employed (Table 5).

**Table 6:** Univariate and multiple logistic regression analysis.

Variables	Univariate Analysis			Multiple Logistic Regression	
	χ^2^	COR (95% CI)	*p-*value	AOD (95% CI)	*p-*value
**Age > 36 years**	7.9688	0.226 (0.069–1.650)	0.033	1.101 (0.650–1.699)	0.003
**Voluntary participation to the STD clinic**	4.4785	1.722 (0.955–2.997)	0.027	1.693 (0.942–2.990)	0.51
**Having symptoms**	5.7627	2.843 (1.228–4.232)	0.015	1.760 (0.254–2.210)	0.113
**Not using condoms at last 3 months**	4.3802	1.182 (0.372–3.830)	0.016	0.819 (0.142–4.295)	0.080
**Not using condom at last sex**	4.3309	2.761 (0.852–10.098)	0.039	2.900 (0.940–8.770)	0.050
**Having sexually transmitted diseases**	5.7573	0.304 (0.002–1.786)	0.001	0.425 (0.254–2.210)	0.120

* COR-crude odd ratio; CI-confidence interval; *p*-value-probability value; AOD-adjusted odd ration.

There was a significant association between the reason for attendance to the STD clinic and trichomoniasis (*p* = 0.027). Three (17.6%) tested positives were referred by the courts suspecting them as commercial sex workers. One trichomoniasis patient was a victim of multiple rape case (1/17, 5.9%). On multiple logistic regression analysis revealed a significantly high infection rate among symptomatic patients who attended clinics voluntarily (OR, 1.693; 95% CI, (0.955–2.997; *p* = 0.051) (Table 6). The Chi-square test (S1 Table) reported a significant association of *Trichomonas* infection with the clinical symptoms (*p* = 0.015). The present study noted that 6 out of 17 tested positives (35.3%) were presented with whitish vaginal discharge at the time of clinical examination. However, the duration of symptoms was not significantly associated with the disease (*p* = 0.864). Among the infected patients, four (23.5%) gave a history of miscarriages and stillbirths. The univariate analysis showed a significant association between the number of sexual partners and *Trichomonas* infection (*p* = 0.001). Of the tested positives, 9 (52.9%) had only a single sexual partner, while the rest revealed multiple sexual relationships (Table 5).

On univariate analysis, a significant association was obtained between condom usage during sex in last three months and trichomoniasis (*p* = 0.016). The majority of the infected patients had never used condoms in last three months (9/17, 52.9%). Furthermore, a significant association was obtained between condom usage at last sexual contact and trichomoniasis (OR = 2.900; 95% CI, 0.940–8.770; *p* = 0.050) (Table 6). Moreover, the study revealed a significant association between other STDs and trichomoniasis (*p* = 0.001). Among the *T*. *vaginalis* positive group, only five (29.4%) patients had trichomoniasis as a single infection, and the rest were co-infected with other STDs (Table 5).

## Discussion

Trichomoniasis is the most common sexually transmitted non-viral disease which affects women of reproductive age and causes severe pregnancy consequences. This infection was underreported due to nonspecific clinical presentation and the nonavailability of sensitive laboratory diagnostic tests in clinical settings [8, 16]. This study was carried out to identify the risk factors associated with *Trichomonas* infection and to compare the sensitivity and specificity of microscopy (wet mount and Giemsa staining), culture, and PCR in the diagnosis of trichomoniasis in Sri Lanka.

The clinical diagnosis based on the signs and symptoms is nonspecific. Therefore, it is important to have a sensitive and specific diagnostic test to diagnose *Trichomonas* infection. In Sri Lanka, the commonly used method for the diagnosis of trichomoniasis in clinical settings is wet mount examination. In the present study, 6 (1.6%) cases were positive for wet mount with a sensitivity of 35.3%, in comparison to PCR. A similar sensitivity (38%) for wet mount examination was reported in Australia [32]. In contrast, some studies have shown that a higher sensitivity for wet mount (60%, 99.2%) compared to PCR [15, 23]. Considering its simplicity and cost-effectiveness, the wet mount is the most commonly used method in clinical settings with limited resources. However, the sensitivity of wet mount is highly dependent on the experience of the microscopist, duration of transport, and laboratory processing before the organism dies or loses its motility [17, 33]. The delays as short as 10–30 min between specimen collection and microscope examination can dramatically reduce the sensitivity of the test. Also, wet mount requires at least 10^4^ organisms per milliliter of vaginal fluids to detect *T*. *vaginalis* [17].

The culture method detected 7 cases (1.8%) with a sensitivity of 41.2% compared to the extended gold standard PCR in the present study. Culture in the anaerobic condition is considered a gold standard for the diagnosis of *T*. *vaginalis* infection for many years [8, 20, 30]. However, culturing is time-consuming [8], and the time of inoculation determines the viability of the organism. If it is not inoculated immediately within one hour of specimen collection, the viability of the organism is lost, and culture becomes ineffective [20, 23, 34]. A density of 10^2^ trichomonads per milliliter is required to culture becomes positive [17]. Although the culture medium is enriched with antibiotics to eliminate vaginal bacteria, contamination with vaginal bacteria is a disadvantage [11].

According to the current study, the genus-specific primer set (TFR1/TFR2) was more sensitive compared to the species-specific primer sets (TV16Sf-2/ TV16Sr-2 and TVK3/TVK7). The highest sensitivity was reported by TV16Sf-2/TV16Sr-2 species-specific primer set (88.2%). In contrast, a previous study reported higher sensitivity of 100.0% and 92.8% for TV16Sf-2/TV16Sr-2 and TVK3/TVK7, respectively [31]. This primer was designed to amplify a DNA sequence in the repetitive DNA of the *T*. *vaginalis* genome, which may vary from strain to strain to cause negative results [23, 31]. Thus, primers targeting conserved regions of the *T*. *vaginalis* genome are recommended for future studies [34, 35]. Compared to microscopy and culture methods, PCR detected more cases, indicating that PCR is a highly sensitive and specific diagnostic technique to be used in resourceful laboratory settings. This has been further recommended by several studies conducted by Patil *et al*. (2012) [23], Crucitti *et al*. (2003) [31], Wendel *et al*. (2002) [36], van Der Schee *et al*. (1999) [37], Mayta *et al*. (2000) [38], and Radonjic *et al*. (2006) [39]. Due to the high detection rate of PCR, the overall prevalence of trichomoniasis reported in this study was 4.4%, which was higher than the prevalence of 2.3% reported by Samarawickrema *et al*. (2015) [27] in Sri Lanka. Similar to the present study, a study carried out by Banneheke *et al*. (2013 b) [40] using the immunochromatographic technique reported a prevalence of 4.2%.

In the present study, the majority of the infected patients were females. It was consistent with the study done by Patel *et al*. (2018) [41] and Tompkins *et al*. (2020) [42] in the USA. High incidence among women was due to the symptomatic infection in females, while the male was mostly asymptomatic, thus transmitting the infection to female partners during unprotected sexual intercourse [43]. Therefore, early diagnosis and treatment of male patients are very important to control the disease.

This study observed age over 36 years was a significant risk factor (*p* = 0.033) associated with the infection. This finding was supported by previous studies [44–46]. Older females are more prone to acquire trichomoniasis due to hormonal changes that affect the vaginal pH level. Similar patterns have also been observed among older men [45].

A significant proportion of infected patients were symptomatic in this study (*p* = 0.015). Vaginal discharge was the most common symptom reported by positive patients for trichomoniasis [14, 25, 47, 48]. When the reasons for STD clinic visits were considered, more than half of the patients had sought medical advice due to the symptomatic nature, and it was significantly associated with the infection (*p* = 0.015). Similar findings were reported by Javanbakht *et al*. (2013) [9]. In Sri Lanka, community-based studies have reported less prevalence than clinic-based studies. This could be due to the high positivity among symptomatic patients who visits STD clinics compared to asymptomatic individuals in the community. Community-based studies carried out by Hemachandra *et al*. (2007) [49] and Herath, (2008) [50] showed a low prevalence (1.0% and 0.6%) of *Trichomonas* infection compared to the clinic-based study carried out by Perera *et al*. (1994) [51] which gave a prevalence of 4.4%. However, some studies have reported a high incidence of asymptomatic infection in men and women [25, 44], while Ambrozio *et al*. (2016) [52] observed the absence of *Trichomonas* infection in the majority of symptomatic women.

Condom usage decreases the risk of trichomoniasis as it acts as a physical barrier and prevents the transmission of the organisms during sexual intercourse. The current study found a significant association between condom usage and trichomoniasis (*p* = 0.016). This is in agreement with the studies of Barbosa *et al*. (2020) and Fernando *et al*. (2012) [46, 53]. The present study reported a statistically significant relationship between *Trichomonas* infection and multiple sexual partners (*p* = 0.001). A similar association was reported in the previous studies carried out in Sri Lanka [14] and in other countries [41, 52].

Studies carried out in Sri Lanka showed that *T*. *vaginalis* infection was not prevalent among commercial sex workers [26, 46]. However, a recent study carried out by Barbosa *et al*. (2020) [53] showed a high prevalence of *Trichomonas* infection among sex workers. The present study observed an increased incidence of infection among commercial sex workers, even though the findings were not statistically significant. The particular reason for the high incidence among commercial sex workers was not clear. *Trichomonas* infected patients were reported from low socioeconomic groups without basic living conditions (e.g. shelter) and engaged in street-based prostitution [42]. Perhaps, the reported high incidence among commercial sex workers may be due to prostitution without proper protective measures (e.g. condoms).

The majority (16/17) of the infected patients in this study group were heterosexual. Although miscarriages/stillbirths were found as complications of trichomoniasis [54], the present study revealed no significant association between *Trichomonas* infection and miscarriages/stillbirth (*p* = 0.762). The present study showed a statistically significant association between *Trichomonas* infection and other sexually transmitted diseases (*p* = 0.001). Inflammation of the vagina caused by *T*. *vaginalis* enhances the susceptibility of patients to human immunodeficiency virus (HIV) infection, chlamydial infection, candidiasis, *Proteus* infection, syphilis, gonorrhea, and herpes simplex virus 1 and 2 infections [55–58]. Also, co-infection of *T*. *vaginalis* with human papillomavirus was reported by Samarawickrema *et al*. (2015) [27] and Barbosa *et al*. (2020) [53].

To date, this is the first study conducted to evaluate the PCR as a diagnostic test for the diagnosis of trichomoniasis in Sri Lanka. Further investigations are required to identify strain variation of *T*. *vaginalis*, which could provide better insight into genetic diversity and the pathogenicity of the organism.

## Conclusions

In conclusion, the present study identified the PCR as a highly sensitive and specific diagnostic test compared to microscopy (wet mount and Giemsa staining) and culture methods suggesting that the importance of setting up PCR-based diagnostic tests for the diagnosis of trichomoniasis in STD clinics in Sri Lanka. Furthermore, the current study identified age over 36 years, symptomatic nature, multiple sexual partners, not using condoms, having other STDs as significant risk factors for contracting *Trichomonas* infection. Thus, screening individuals above 36 years for trichomoniasis, incorporating sex education into formal education, and implementing public awareness programmes on safe sex are recommended.

## Supporting information

S1 Table Statistical analysis. Chi-square test and Fisher’s test.
(DOCX)

S1 Fig Light microscopic view of *Trichomonas* trophozoites. A: *Trichomonas trophozoites* in wet mount method (x40), arrow shows motile pear-shaped trophozoites. B: *Trichomonas trophozoites* in Giemsa staining method, arrow shows pear-shaped trophozoites.
(TIF)

S1 Raw images Raw images of agarose gel and microscopic slides. (PDF)

S1 Appendix Calculation of sample size. (DOC)

S2 Appendix The consent form and research questionnaire. (PDF)

S3 Appendix Calculation of expanded gold standard. Comparison of the wet mount, Giemsa staining, culture, PCR using TFR1/2, TV16Sf/r, and TVK3/7 and expanded gold standard.
(DOCX)
