# Supplementary material for: Comparison of diagnostic methods and analysis of socio-demographic factors associated with Trichomonas vaginalis infection in Sri Lanka
Source: PLoS One. 2021 Oct 13;16(10):e0258556. doi: 10.1371/journal.pone.0258556 (PMC8513885; doi:10.1371/journal.pone.0258556)
Supplement: S1 Table — Chi-square test and Fisher’s test. (DOCX) [file pone.0258556.s001.docx]

**Table 1:** Association between socio-demographic factors and the trichomoniasis in Sri Lanka.

|  | **Variable** | **Chi-Squared Test** | **Fisher’s Test** |
| --- | --- | --- | --- |
|  |  | **p-value** | **p-value** |
| 1 | Gender | 0.24190 | 0.18840 |
| 2 | Civil status | 0.08497 | 0.06167* |
| 3 | Age | 0.04037 | 0.03317** |
| 4 | District | 0.50630 | 0.41100 |
| 5 | Province | 0.27430 | 0.25580 |
| 6 | level of education | 0.58690 | 0.28250 |
| 7 | Job | 0.13940 | 0.13440 |
| 8 | Reason for attendance | 0.00015 | 0.02713** |
| 9 | Symptoms | 0.01474** | 0.26570 |
| 10 | Duration of symptoms | 0.94870 | 0.86450 |
| 11 | medication | 0.56950 | 0.32000 |
| 12 | miscarriage/still birth | 1.00000 | 0.76260 |
| 13 | sex contact | 0.83900 | 0.86140 |
| 14 | Type of partner | 0.46120 | 0.36410 |
| 15 | Sexual orientation | 0.79340 | 0.90650 |
| 16 | No of partner | 0.00000 | 0.00183** |
| 17 | Condom use at last sex | 0.50060 | 0.42240 |
| 18 | Condom use at last 3 months | 0.26970 | 0.22790 |
| 19 | Substace abuse | 0.34930 | 0.36870 |
| 20 | Previous STD | 0.24240 | 0.14810 |
| 21 | Age at 1st sex | 0.08031 | 0.08652* |
| 22 | Total No of partners last 3 months | 0.00310 | 0.01311** |
| 23 | Total No of partners last I year | 0.07393 | 0.06728* |
| 24 | Total No of partners life time | 0.17670 | 0.13200 |
| 25 | Signs | 0.89990 | 0.72200 |
| 26 | probable dx | 1.101e-15** | 0.00140 |

** and * were shown significant p-values.

Total of 26 socio-demographic factors were analysed in the current study. The association between these socio-demographic factors and the trichomoniasis disease in Sri Lanka was established using the two statistical tests called Pearson’s Chi-square test and Fisher’s exact test. Since some of the combinations of variables did not follow the all parametric assumptions, for those combination of variables, Fisher’s exact test was used to determine the relationship.

Age (p-value 0.03317), reason for attendance (p-value 0.02713), symptoms (p-value 0.01474), no. of partners (p-value 0.00183), total no. of partners at last 3 months (p-value 0.01311), and probable dx (p-value 1.101e-15) were shown a relationship to trichomoniasis disease (p<0.05) (Table 1).

Civil status of the patient (p-value 0.06167), age at 1^st^ sex (p-value 0.08652) and total no. of partners at last one year (p-value 0.06728) were also reported p-values closer to 0.05, suggesting that these factors were also associated with trichomoniasis disease.

However, the other factors (Table 1) were shown larger p-values compared to the significant level (p>0.05). Thus, there was no enough evidence to reject the null hypothesis, which suggesting that those factors were not associated with trichomoniasis disease.
