# Supplementary figures and images for: Comparison of diagnostic methods and analysis of socio-demographic factors associated with Trichomonas vaginalis infection in Sri Lanka
Source: PLoS One. 2021 Oct 13;16(10):e0258556. doi: 10.1371/journal.pone.0258556 (PMC8513885; doi:10.1371/journal.pone.0258556)

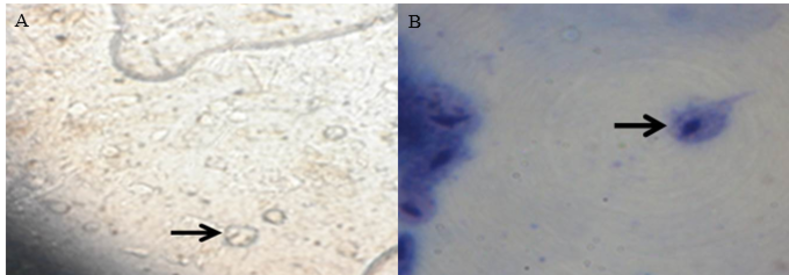

Supplement: S1 Fig — A: Trichomonas trophozoites in wet mount method (x40), arrow shows motile pear-shaped trophozoites. B: Trichomonas trophozoites in Giemsa staining method, arrow shows pear-shaped trophozoites. (TIF) [file pone.0258556.s002.tif]

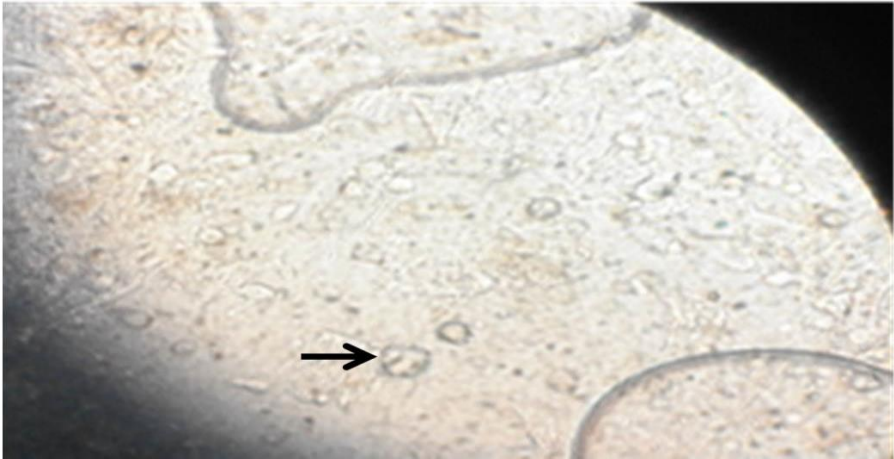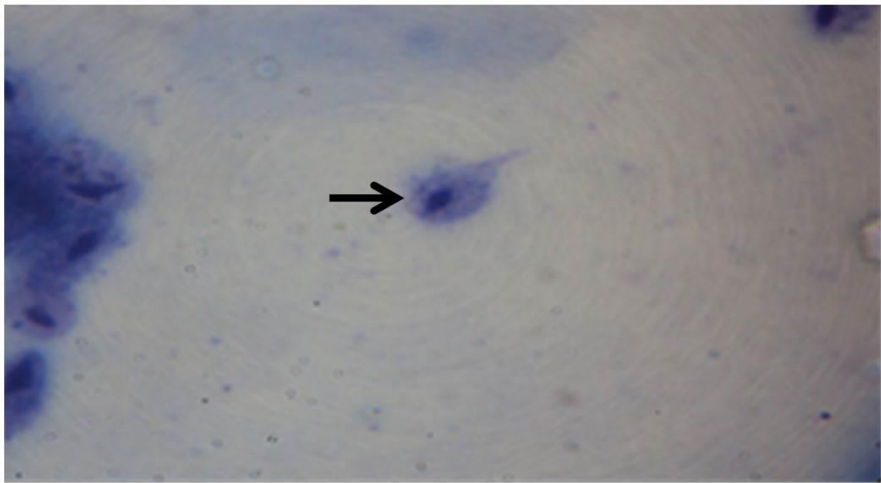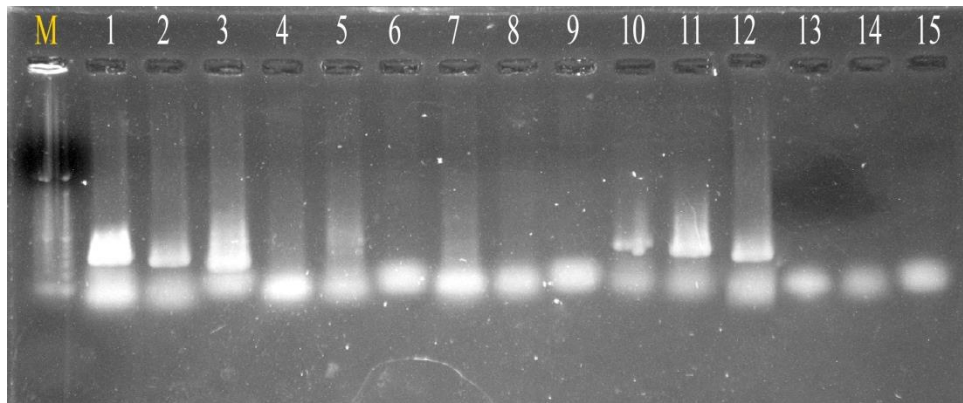

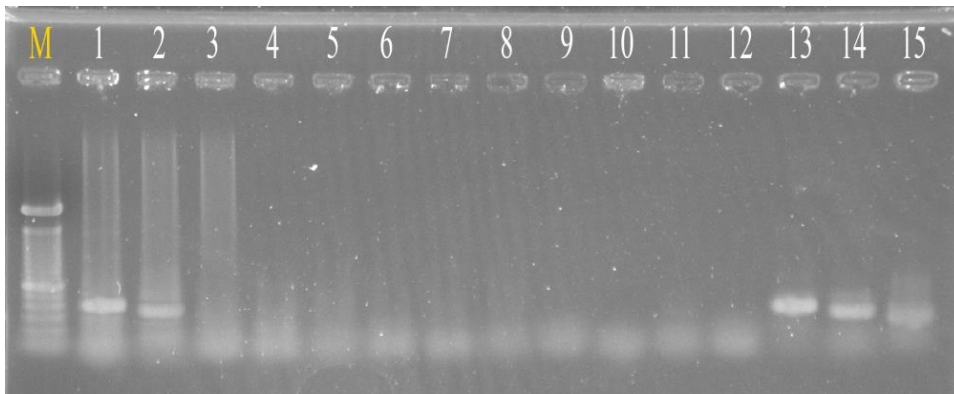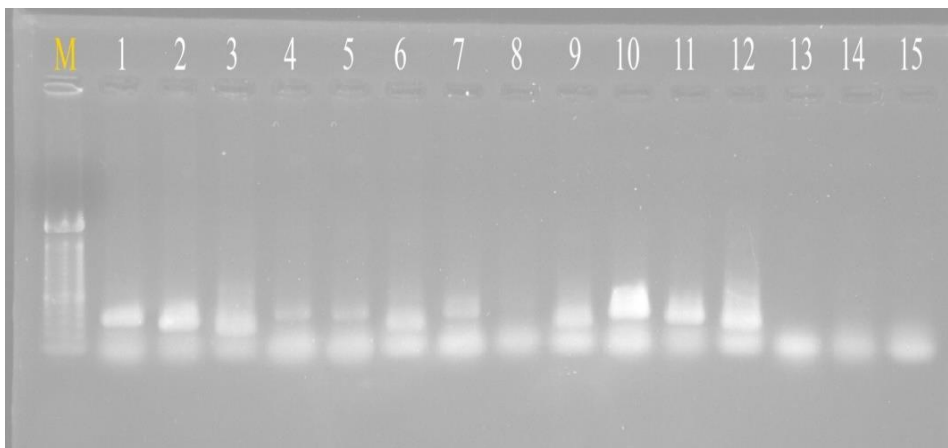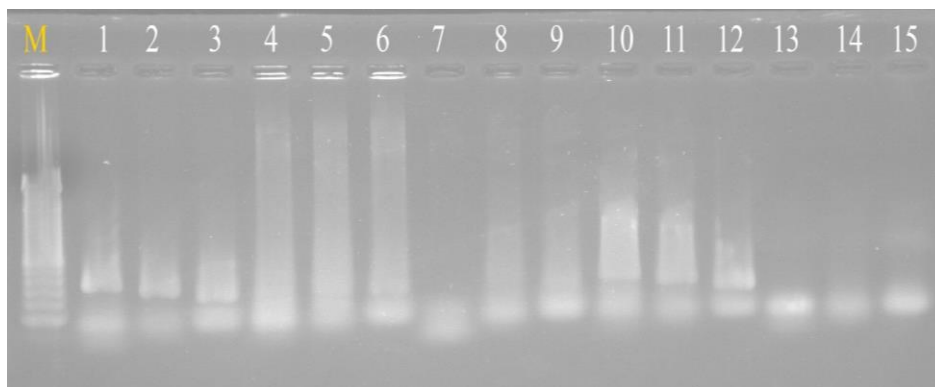

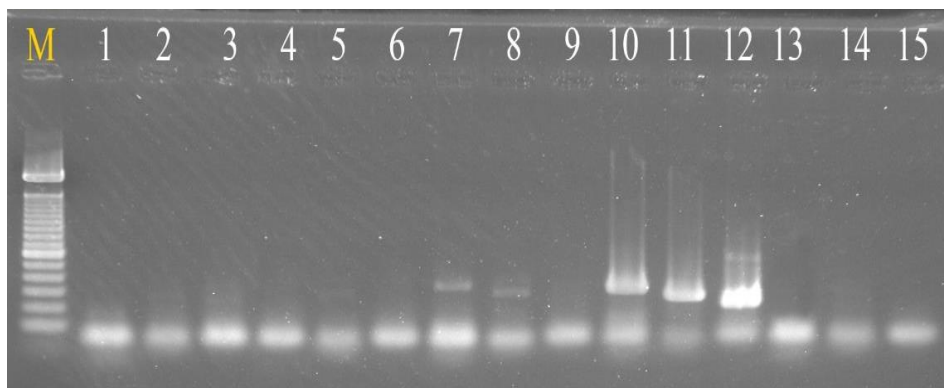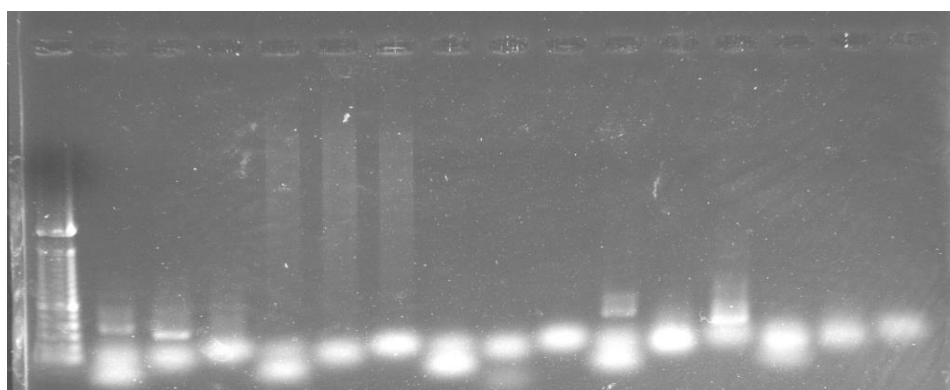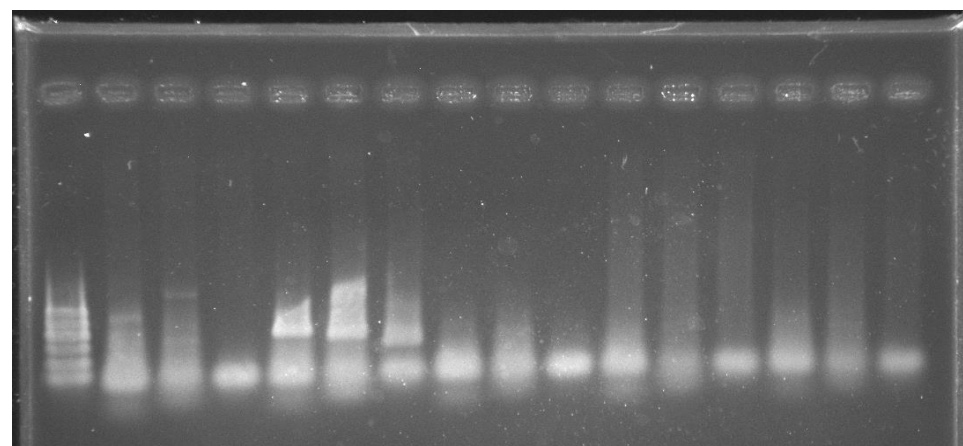

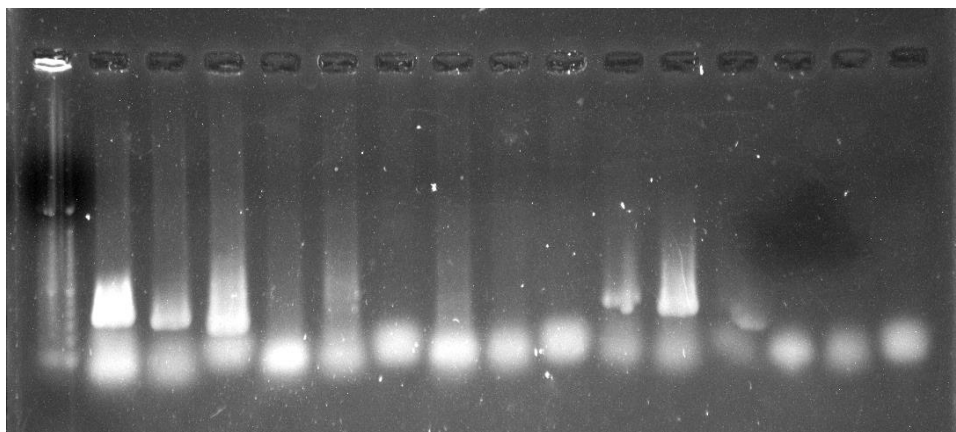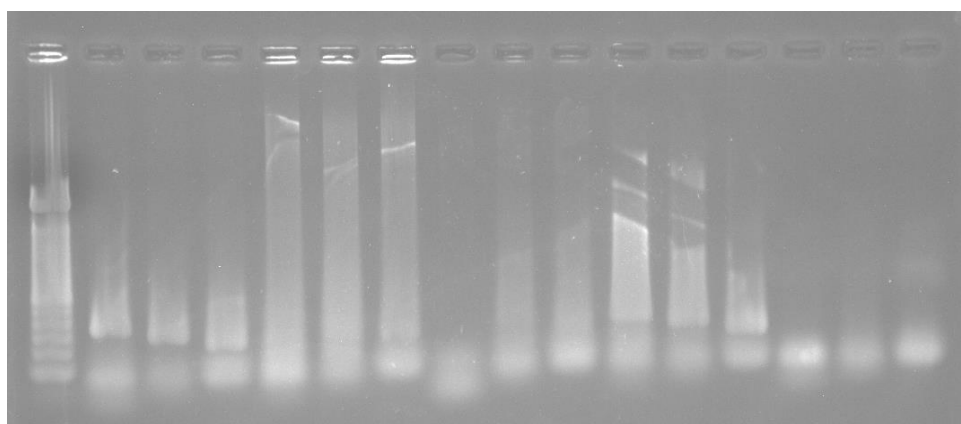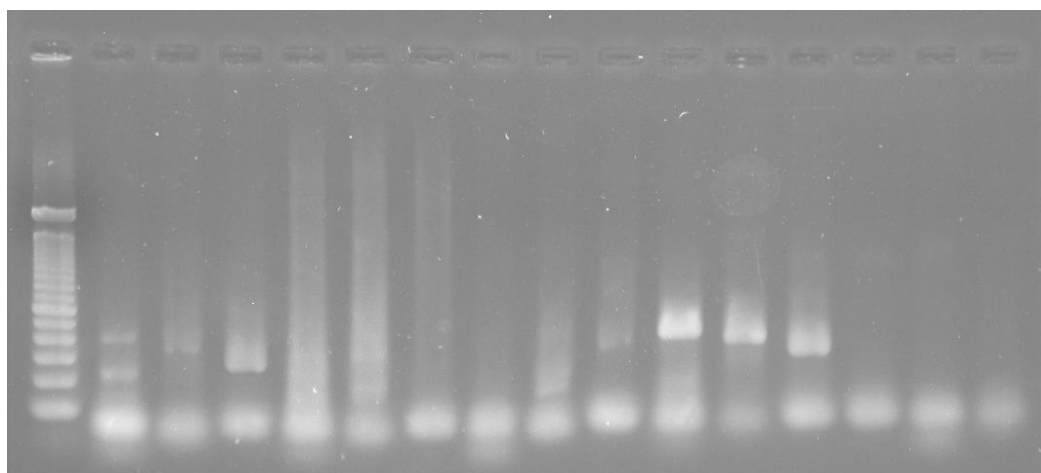

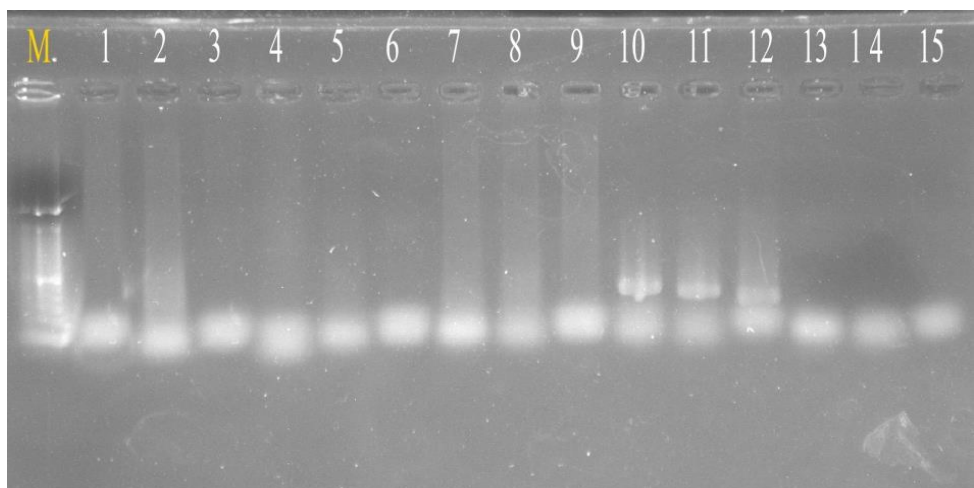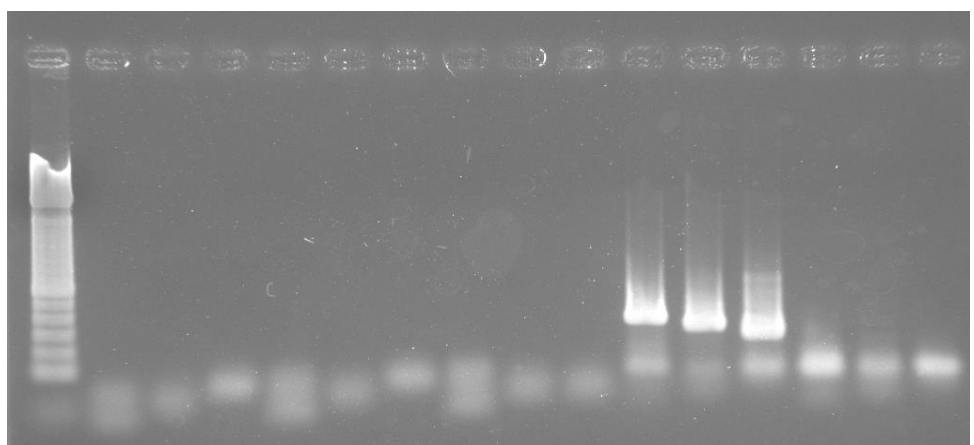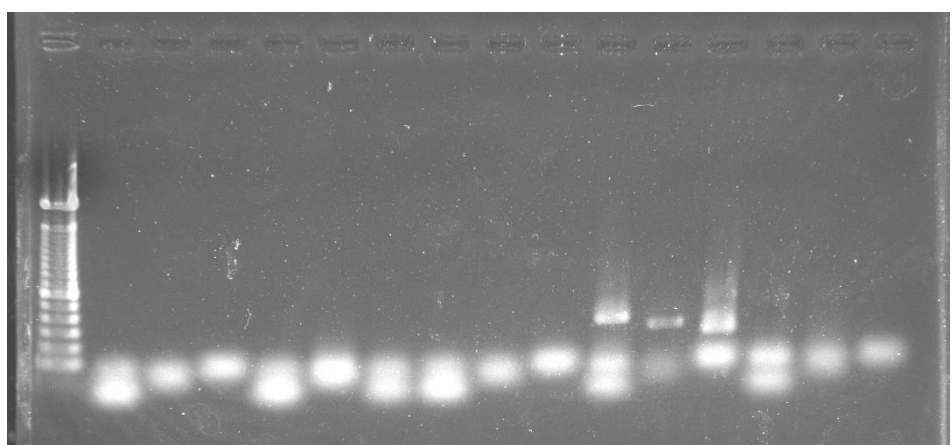

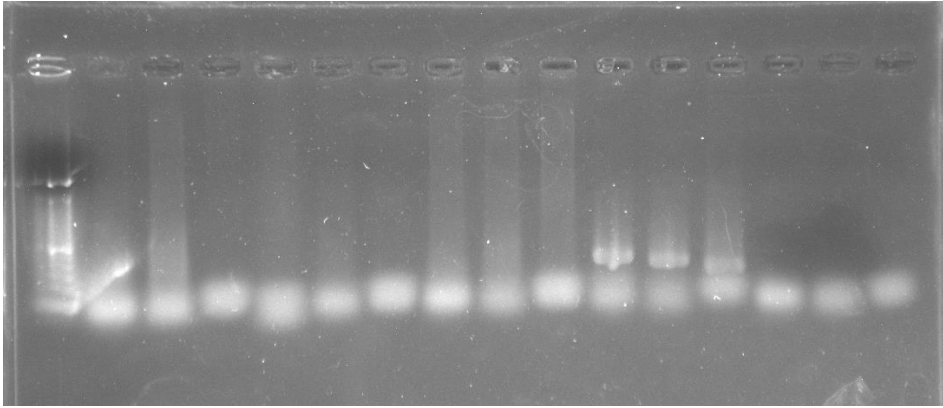

Supplement: S1 Raw images — (PDF) [file pone.0258556.s003.pdf]
