# Supplementary material for: Comparison of diagnostic methods and analysis of socio-demographic factors associated with Trichomonas vaginalis infection in Sri Lanka
Source: PLoS One. 2021 Oct 13;16(10):e0258556. doi: 10.1371/journal.pone.0258556 (PMC8513885; doi:10.1371/journal.pone.0258556)
Supplement: S1 Appendix — (DOC) [file pone.0258556.s004.doc]

**Calculation of sample size**

Sample size (n) required for the study was calculated using the following equation (Charan and Biswas, 2013).

n = Z _1-α/2_ ^2^ p (1 – p)

d^2^

Z _1-α/2_ = Standard normal variate (at 5% type 1 error [*P*<0.05] it is 1.96)

p = Expected proportion in population based on previous studies

d = Absolute error or precision

n = 1.96 ^2^ x 0.72 (1 – 0.72)

0.05^2^

n= 309.78

n= 310.00

Hence, required sample size for the study is 310. However, to increase the power of the study, 385 samples were collected during the study period.
