# Supplementary material for: Comparison of diagnostic methods and analysis of socio-demographic factors associated with Trichomonas vaginalis infection in Sri Lanka
Source: PLoS One. 2021 Oct 13;16(10):e0258556. doi: 10.1371/journal.pone.0258556 (PMC8513885; doi:10.1371/journal.pone.0258556)
Supplement: S2 Appendix — (PDF) [file pone.0258556.s005.pdf]

Patients consent forms

පර්යේෂණය සඳහා අනුමැතිය ලබා දිමේ ප්‍රකාශය

විද්‍යාත්මක පර්යේෂණය : ට්‍රයිකොමොනාසිස් නම් වු රෝගය හඳුනා ගැනිම සඳහා PCR පරීක්ෂාව ගොඩ නැගීම සහ ට්‍රයිකොමොනාස් වැජිනාලිස් ජීවියාගේ අණුක ගති ලක්‍ෂණ සොයා බැලිම.

මෙම අනුමැතිය දිමේ ප්‍රකාශය අදාල වන්නේ ඉහත මාතෘකාවේ සඳහන් විද්‍යාත්මක පරික්‍ෂණය සඳහා සහභාගි වන්නන් සඳහාය

ප්‍රධාන පර්යේෂක : වෛද්‍ය ඩබ්.එම්.ඩී.ආර් ඉද්දවෙල
ජ්‍යෙෂ්ඨ කථිකාචාර්ය
පරපෝෂිතවේදි දෙපාර්තමේන්තුව
වෛද්‍ය පීඨය
පේරාදෙණිය විශ්වවිද්‍යාලය

අනෙකුත් පර්යේෂකයන් : එච්.එම්.එස්.පී. හේරත්
පරපෝෂිතවේදි දෙපාර්තමේන්තුව
වෛද්‍ය පීඨය
පේරාදෙණිය විශ්වවිද්‍යාලය

වෛද්‍ය සුසිජි වික්‍රමසිංහ
අංශ ප්‍රධානී/ජ්‍යෙෂ්ඨ කථිකාචාර්ය
පරපෝෂිතවේදි දෙපාර්තමේන්තුව
වෛද්‍ය පීඨය
පේරාදෙණිය විශ්වවිද්‍යාලය

වෛද්‍ය ගංගා පතිරණ
ලිංගාශ්‍රිත රෝග පිළිබඳ විශේෂඥ වෛද්‍ය
ලිංගාශ්‍රිත රෝග හා ඒඩ්ස් නිවාරණ ඒකකය

මෙම අනුමැතිය දිමේ ප්‍රකාශය කොටස් දෙකකින් යුක්තය.

1. දැනුවත් කිරිමේ පත්‍රිකාව
2. අනුමැතිය ලබා දිමේ පත්‍රිකාව

PART I - දැනුවත් කිරිමේ පත්‍රිකාව

1. හැඳින්වීම.

පේරාදෙණිය විශ්වවිද්‍යාලයේ වෛද්‍ය පීඨයේ පරපෝෂිතවේදි දෙපාර්තමේන්තුව හි සේවය කරන අපි ඉහත සඳහන් පර්යේෂණය කිරිමට බලාපොරොත්තු වෙමු. මෙම මාතෘකාව වෛද්‍යමය/සෞඛ්‍යමය වශයෙන් වැදගත්ය. මෙම පර්යේෂණය සඳහා ඔබට සහභාගි විමටත්, ඒ පිළිබඳව දැනුවත් විමටත් ආරාධනා කරමු. මේ සඳහා වු තීරණය දැන් ලබාදිම අවශ්‍යය නොවේ. මේ පිළිබඳව ඔබ වෙනත් දැනුවත්තෙකු සමඟ හෝ සාකච්ජා කර නිවැරදි තිරණයකට එළඹෙන ලෙස ඉල්ලා සිටිමු. මෙම පත්‍රිකාවෙහි නොතේරුණු යමක් වේ නම් ඒ පිළිබඳව විමසන මෙන් ඉල්ලා සිටිමු.

2. පර්යේෂණයේ අරමුණ.

§*ට්‍රයිකොමොනාසිස්*¶ ලිංගිකව සම්ප්‍රේෂණය වන රෝගයකි. එය *ට්‍රයිකොමොනාස් වැජිනාලිස්* නම් වු රෝග කාරකයෙන් හට ගන්නකි. රෝගියා පෙන්නුම් කරන රෝග ලක්‍ෂණයන්ට අනුව මෙම රෝගය නිවැරදිව හඳුනාගැනිම අසීරු වේ. එබැවින් රෝගය නිවැරදිව හඳුනාගැනිම සඳහා රසායනාගාර පරික්‍ෂණ යොදා ගැනිම ඉතා වැදගත් වේ.

ට්‍රයිකොමොනාසිස් රෝගය හඳුනා ගැනීම සඳහා රසායනාගාර පරීක්ෂණ කිහිපයක් දැනට භාවිතා වන අතර, ඒ සඳහා වඩාත්ම නිරවද්‍ය ලෙස සැලකෙන PCR ක්‍රමය ශ්‍රී ලංකාව තුල ගොඩනැගීම මෙම පර්යේෂණයේ ප්‍රධාන පරමාර්ථය වේ.

3. පර්යේෂණයේ ස්වභාවය.

මෙම පර්යේෂණය සඳහා ලිංගාශ්‍රිත රෝග හා ඒඩ්ස් රෝගය නිවාරණ ඒකකයට පැමිණෙන ස්ත්‍රී/පුරුෂ ඔබ දෙපාර්ශ්වයම යොදා ගැනීමට අපේක්ෂා කරමු. එහිදී ඔබගේ විස්තර හා රෝග ඉතිහාසය ලබා ගනිනු ඇත. එයට අමතරව යෝනි මාර්ගයෙන් / මුත්‍රා මාර්ගයෙන් සාම්පලයක් (*four vaginal l urethral swabs*) සහ මුත්‍රා සාම්පලයක් ලබා ගනු ඇත.

4. සහභාගි වන්නන් තෝරා ගැනීමේ ක්‍රියාමාර්ග.

ඔබ වයස අවුරුදු 15 - 55 ත් අතර වයස, කිසියම් වූ ලිංගාශ්‍රිත රෝගයකින් පෙළේ යැයි සැක සහිත නම් මෙම පර්යේෂණය සඳහා සහභාගි විය හැක.

එහෙත් ඔබ මෙම පරීක්ෂාවට සති දෙකක් ඇතුලත *Metranidazole* බෙහෙත් පෙති වර්ගය ලබාගෙන ඇත්නම් මේ සඳහා සහභාගි නොවන ලෙස කාරුණිකව ඉල්ලා සිටිමු.

5. සහභාගිත්වය ස්වේච්ඡාවෙන්ම බව.

ඔබගේ මෙම සහභාගී වීම සම්පූර්ණයෙන්ම ස්වේච්ඡා සහභාගී වීමක් වේ. සහභාගී වීම හෝ නොවීම ඔබ විසින්ම තිරණය කළ යුතුයි. මෙම තිරණය ඔබගේ ප්‍රතිකාර සැපයීම සඳහා කිසිඳු බලපෑමක් නොවන බව සහතික කරමු.

6. පරීක්ෂණ ක්‍රියාපටිපාටිය.

පර්යේෂණය සඳහා ඔබගේ විස්තර (ජීව දත්ත) හා රෝග ඉතිහාසයට අමතරව යෝනි මාර්ගයෙන් / මුත්‍රා මාර්ගයෙන් සාම්පලයක් (*four vagianl / urethral swabs*) සහ මුත්‍රා සාම්පලයක් ලබා ගනිනු ඇත. මෙහිදී ජීවාණුහරණයට අදාල සම්මත පූර්ව ආරක්ෂණ ක්‍රම සියල්ල භාවිතා කරනු ඇත. තවද ඔබගේ පෞද්ගලිකත්වයට කිසිඳු හානියක් නොවන ලෙස කටයුතු කරන අතර, සියළු තොරතුරු නිර්නාමිකව ලබා ගැනේ. එකතු කරගත් සාම්පල භාවිතාකර,

- *Wet mount preparation*
- *Permanent staining*
- *Culture*
- *PCR*

යන රසායනාගාර පරීක්ෂා සිදු කිරීමට බලාපොරොත්තු වෙමු. අවසානයේදී PCR ක්‍රමය සහ Culture ක්‍රමයෙහි ප්‍රතිඵල සංසන්දනය කරනු ඇත.

මෙම පරීක්ෂණය අවසානයේ ඉතිරිවන සාම්පල සියල්ල විනාශ කරනු ඇත.

7. පර්යේෂණ කාලය

මෙම පර්යේෂණය 2017/04/01 සිට මාස 24ක කාලයක් පුරා සිදු කෙරේ.

8. අතුරු ආබාධ.

මෙම පර්යේෂණයට සහභාගී වීමෙන් ඔබට කිසිඳු අතුරු ආබාධයක් ඇති නොවන බව අප අවධාරණය කරමු. මන්ද, සායනයේදී සාමාන්‍යයෙන් සිදු කරන ක්‍රියා පටිපාටියන්ට අමතරව කිසිඳු පියවරක් අප විසින් සිදුකරනු නොලැබේ.

9. ප්‍රතිලාභ.

ඔබ මෙම පර්යේෂණයට සහභාගි වුවහොත් සාම්පලය ලබාගත් දින සිට මාසයක් ඇතුලත අප විසින් සිදුකරන ලද පරීක්ෂාවන්ට අදාල වාර්ථාව ඔබ වෙත ලබා දීමට කටයුතු සකස් කරන අතර, ඊට අමතරව ට්‍රයිකොමොනාසිස් රෝගය හඳුනා ගැනීම සඳහා වැඩි දියුණු කරන ලද PCR ක්‍රමය ශ්‍රී ලංකාව තුල හඳුන්වා දීමට හැකියාව ලැබේ.

10. මුදල් ප්‍රතිපූරණය.

මෙම පර්යේෂණයට සහභාගිවීම සඳහා දිරි ගැන්වීමේ දීමනාවක් දෙනු නොලැබේ.

11. තොරතුරුවල රහස්‍යභාවය.

මෙම පර්යේෂණයේදී ඔබගෙන් ලබා ගන්නා තොරතුරු වල රහසිගත බව තරයේ ආරක්ෂා කරන බවට සහතික වෙමු.

12. ප්‍රතික්ෂේප කිරීමට ඇති අයිතිය.

පර්යේෂණ සඳහා ඔබගේ සහභාගීත්වය සම්පූර්ණයෙන්ම ඔබගේ ස්වෙච්ජාවෙනි. ඕනෑම අවස්ථාවක සහභාගීත්වයෙන් ඉවත්වීමට සම්පූර්ණ නිදහස ඔබට ඇත.

13. තොරතුරු දැනගැනීම සඳහා සම්බන්ධීකරණය.

ඔබ මේ පර්යේෂණය අතරමග ඕනෑම මොහොතකදී යම් තොරතුරක් දැනගැනීමට අවශ්‍යවන්නේ නම් පහත සඳහන් දුරකථන / විද්‍යුත් ලිපිනයන් මාර්ගයෙන් අප සම්බන්ධ කර ගන්නා මෙන් ඉල්ලා සිටිමු.

- ❖ වෛද්‍ය ඩබ්.එම්.ඩී.ආර් ඉද්දවෙල
  ජ්‍යෙෂ්ඨ කථිකාචාර්ය
  පරපෝෂිතවේදි දෙපාර්තමේන්තුව
  වෛද්‍ය පීඨය
  පේරාදෙණිය විශ්වවිද්‍යාලය

- ❖ එච්.එම්.එස්.පී. හේරත්
  පරපෝෂිතවේදි දෙපාර්තමේන්තුව
  වෛද්‍ය පීඨය
  පේරාදෙණිය විශ්වවිද්‍යාලය

මෙම පර්යේෂණ යෝජනාව පේරාදෙණිය වෛද්‍ය පීඨයේ මානව ආචාරධර්ම කමිටුව මගින් සමාලෝචනය කර අනුමත කර ඇත. පේරාදෙණිය වෛද්‍ය පීඨය මානව ආචාරධර්ම කමිටුව පර්යේෂණයන් සඳහා සහභාගී වන අයගේ ආරක්ෂාව තහවුරු කිරීමට පිහිටුවා ඇත. මේ පිළිබඳ ඔබට තව දුරටත් දැන ගැනීමට කැමති නම් දුරකථන අංක 0717555495 ට කථා කරන්න.

PART II - අවසර පත්‍රය

මම ඉහත තොරතුරු පත්‍රිකාව මනාව කියවා තේරුම් ගතිමි. සමහර නොතේරෙන අවස්ථාවලදී ඒ පිළිබඳව ප්‍රශ්න අසා හොඳින් තේරුම් ගතිමි. මම ස්වෙච්ඡාවෙන් මෙම පර්යේෂණය සඳහා සහභාගී වීමට කැමැත්ත ප්‍රකාශ කරමි.

සහභාගි වන්නාට අදාල අංකය (Serial No): ______

සහභාගි වන්නාගේ ඇඟිලි සළකුණ ______

දිනය ______

ஒப்புதல் பத்திரம்

விஞ்ஞான ஆராய்ச்சி:-

''ட்ரைக்கோமொனாசிஸ்''எனும் நோயை அடையாளம் காண்பது தொடர்பாக PCRபரிசோதனை மேற்கொள்ளலும் ட்ரைக்கோமொனாசிஸ் வெஜினாலிஸ் நோயாளியின் நோய்க்கான அறிகுறிகளை கண்டறிதலும்,

இவ் ஒப்பதல் பத்திரம் மேற் கூறப்பட்ட தலைப்பின் கீழ் இரசாயண பரிசோதனை மேற்கொள்வதற்கு பங்கு பற்றுவோருக்கு,

பிரதானஆராய்ச்சியாளர் :- Dr. W. M. D. R.இத்தவெல,
பிரதான விரிவுரையாளர்,
ஒட்டுண்ணியியல் திணைக்களம்,
வைத்திய பீடம்,
பேராதனைப் பல்கலைக்கழகம்.

ஏனைய ஆராய்ச்சியாளர்கள்:- Ms. H. M. S. P. ஹேரத்,
ஒட்டுண்ணியியல் திணைக்களம்,
வைத்திய பீடம்,
பேராதனைப் பல்கலைக்கழகம்.

பகுதி சார் தலைமை விரிவுரையாளர்:- Dr. S. விக்கிரமசிங்க,
ஒட்டுண்ணியியல் திணைக்களம்,
வைத்திய பீடம்,
பேராதனைப் பல்கலைக்கழகம்.

ஆலோசகர்:- Dr. G. பத்திரன,
பாலியல் நோய் சம்பந்தமான விஷேட
பாலியல் நோய் மற்றும் எயிட்ஸ் நிவாரணப்
பிரிவு, கண்டி.

இவ் ஒப்புதல் பத்திரம் இரண்டு பிரிவுகளாகப் பிரிக்கப்பட்டுள்ளது.

1) தகவல் தெரிவிப்புப் பத்திரம்
2) அனுமதி பத்திரம்

Part I - தகவல் தெரிவிப்புப் பத்திரம்

அறிமுகம் :-

பேராதனைப் பல்கலைக்கழக வைத்திய பீடத்தில்,ஒட்டுண்ணியியல் திணைக்களத்தில் சேவை புரியும் நாம், மேற்கூரிய பரிசோதனையை மேற்கொள்வதற்கு எதிர்ப்பார்க்கின்றோம். இத்தலைப்பு வைத்திய மற்றும் சுகாதாரம் தொடர்பாக மிக முக்கியமானது. இப்பரிசோதனை தொடர்பாக தாங்கள் கலந்து கொள்வதற்கும், இது

தொடர்பான விழிப்புணர்வை பெற்றுக்கொள்வதற்கும் தாங்களை வரவேற்கின்றோம். இது தொடர்பான தீர்மானங்களை உடனடியாக மேற்கொள்ள வேண்டிய அவசியமில்லை. இது தொடர்பாக தாங்கள் வேறு ஒரு ஆலோசகரிடம் அல்லது நபருடன் கலந்துரையாடி சரியான தீர்மானங்களை மேற்கொள்ளுமாறு கேட்டுக்கொள்கின்றோம்.இப்பத்திரத்தில் தாங்களுக்கு புரியாதவற்றை இங்கு கேட்டு தெழிவு பெறலாம்.

ஆராய்ச்சியின் நோக்கம் :-

“ட்ரைக்கோமொனாசிஸ்” என்பது பாலியல் தொடர்புடன் சம்பந்தப்பட்ட ஒரு நோயாகும். இது “ட்ரைக்கோமொனஸ் வெஜினலிஸ்”என்பதிலிருந்து ஏற்படுகின்றது. நோயாளர்கள் காண்பிக்கும் நோய் அறிகுறிகளைக் கொண்டு இந்நோயை சரியான முறையில் கண்டு பிடிப்பது சிறந்ததாகும். இருந்தும் நோயை சரியான முறையில் அறிந்து கொள்ள இரசாயண பரிசோதனை மேற்கொள்வது மிக முக்கியமாகும்.

“ட்ரைக்கோமொனாசிஸ்” நோயை அறிந்து கொள்வதற்கான இரசாயண பரிசோதனைகள் தற்போது நடைமுறையில் உள்ளது. இதில் PCR முறையே மிகவும் சரியான முறை முதன்முறையாக இலங்கையில் மதிப்பீடு செய்யப்பட்டுள்ளது.

ஆராய்ச்சியின் வகை :-

பாலியல் நோய் மற்றும் எயிட்ஸ் நோய் நிவாரணப்பிரிவு, கண்டி. இங்கு வருகை தந்திருக்கும் ஆண் பெண் இரு பாலாருக்கும் இந்நோய்க்கான பரிசோதனையை மேற்கொள்ள முடியும். அதன் போது தங்கள் விபரம் மற்றும் நோய் வரலாறு என்பன பெற்றுக்கொள்ளப்படும். மேலும் தாங்களிடம் யோனி வழியாக/சிறுநீர் வழியாக மாதிரி (Sample) ஒன்றும் (Four Vaginal/Urethral Swabs), சிறுநீர் மாதிரியொன்றும் பரிசோதனைக்காக பெற்றுக்கொள்ளப்படும்.

பங்கு கொள்வோர் தெரிவு செய்யும் முறை :-

தாங்கள் 15 – 55 இடைப்பட்டவராகவும் பாலியல் நோய் தொடர்பாக ஏதாவது நோய் அறிகுறிகள் இருப்பதாக சந்தேகிப்பவர்கள் இப்பரிசோதனையில் கலந்து கொள்ளலாம்.

அதே நேரம் தாங்கள் கடந்த இரு வாரங்களுக்கிடையில் Metronidazole மருந்து உட்கொண்டவராக இருந்தால் தயவு செய்து இப் பரிசோதனை மேற்கொள்ள வேண்டாம் என்று கேட்டுக்கொள்கின்றோம்.

பங்கு கொள்வோர் தெரிவு :-

தாங்கள் பங்கு கொள்வது முற்றிலும் தாங்களது சொந்த தெரிவாகும். பங்கு கொள்வதும் கொள்ளாததும் தங்கள் விருப்பமாகும். இந்த தீர்மானத்தில் தங்களுக்கு சிகிச்சை வழங்குவது தொடர்பாக எந்த கட்டாயமும் இல்லை என்பதை உறுதிப்படுத்துகின்றோம்.

பரிசோதனை நடைமுறைகள் மற்றும் நெறிமுறை :-

இப்பரிசோதனைக்காக தங்களது விபரம் மற்றும் நோய் வரலாறு என்பன தவிர மேலும் தாங்களிடம் யோனி வழியாக/சிறுநீர் வழியாக மாதிரி (Sample) ஒன்றும் (Four Vaginal/Urethral Swabs), சிறுநீர் மாதிரியொன்றும் பரிசோதனைக்காக பெற்றுக்கொள்ளப்படும். இங்கு ஒவ்வொரு நபரினதும் மாதிரிகள் மரபணு பாதுகாப்புக்கறுதி தனிமைப்படுத்தப்பட்ட முறையிலேயே பரிசோதனைக்கு உட்படுத்தப்படும். மேலும் தங்களது தனிப்பட்ட வாழ்க்கைக்கு எந்த பிரச்சினையும்

ஏற்படாத முறையில் நடைமுறைப்படுத்தப்படும் என்பதுடன், அனைத்து விபரங்களும் தனிப்பட்ட முறையில் பேணப்படும் என்பதை அறியத்தருகின்றோம். ஒன்று திரட்டிய மாதிரிகளைப் பயன்படுத்தி,

- Wet mountain preparation
- Permanent staining
- Culture
- PCR

இவ் இரசாயன பரிசோதனைகளை மேற்கொள்ள எதிர்ப்பார்க்கின்றோம். இறுதியில் PCR முறை மற்றும் Culture முறையில் முடிவுகள் தெரியப்படுத்தப்படும்.

இப்பரிசோதனை முடிவில் மிகுதியாகும் மாதிரிகள் முற்றிலும் அழிக்கப்படும்.

பரிசோதனைக்காலம் :-

இப்பரிசோதனை 2017.04.01 முதல் 24 மாதங்களுக்கு மேற்கொள்ளப்படும்.

அபாய நேர்வு(Risk) :-

இப்பரிசோதனையில் கலந்து கொள்வதில் தாங்களுக்கு எந்த வித அபாயங்களும் ஏற்படாமல் நாம் அவதானம் எடுப்போம் என்பதுடன் மருந்தகத்தில் (Clinic)தாங்கள் சாதாரணமான முறையில் நடாத்தப்படுவீர்கள். மேலதிகமாக எந்த வித நெறிமுறைகளும் எம்மால் மேற்கொள்ளப்படமாட்டாது என்பதை அறியத்தருகின்றோம்.

நன்மைகள் :-

தாங்கள் இப்பரிசோதனையில் பங்கு கொண்டால் மாதிரிகளைப் பெற்றுக்கொள்ளும் நாள் தொடக்கம் ஒரு மாதத்திற்குள் எம்மால் மேற்கொள்ளப்படும் பரிசோதனைகளுக்கான முடிவுகளை தங்களிடம் பெற்றுத்தர முடியும் என்பதுடன் மேலும் ''ட்ரைக்கோமொனாசிஸ்'' நோயை அறிந்து கொள்வது தொடர்பாக முன்னேற்றமடைந்துள்ளPCR பரிசோதனை முறையை இலங்கையில் அறிமுகப்படுத்த முடியுமாக இருக்கும்.

பணக் கொடுப்பனவு :-

இப்பரிசோதனையில் கலந்து கொள்வதற்கு தாங்கள் எந்த விதமான பணச்செலவுகளும் செய்யவேண்டியதில்லை.

இரகசியத்தன்மை :-

இப்பரிசோதனைக்காக தங்களிடமிருந்து பெற்றுக்கொள்ளப்படும் தகவல் தொடர்பாக இரகசியம் பேணப்படும் என்பதை உறுதிப்படுத்துகின்றோம்.

மறுப்பிற்கான உரிமை :-

இப்பரிசோதனைக்கான தங்கள் பங்களிப்பு முழுவதும் தங்கள் சுய தீர்மானமாக இருக்க வேண்டும். வேண்டிய நேரத்தில் மறுப்பு தெரிவிப்பதற்கான சகல உரிமைகளும் தங்களுக்கு உள்ளது.

தகவல் அறிய :-

நீங்கள் இப்பரிசோதனை நடைபெறும் காலத்தில் ஏதாவது ஒரு சந்தர்ப்பத்தில் ஏதும் தகவல் தெரிய வேண்டுமானால் கீழ் காணும் ஏதாவது ஒரு முகவரியுடன் அல்லது தொலைபேசியினூடாக தொடர்பு கொள்ள முடியும்.

பெயர் :- Dr. W. M. D. R. இத்தவெல,
முகவரி :- ஒட்டுண்ணியியல் திணைக்களம், வைத்திய பீடம்,
பேராதனைப் பல்கலைக்கழகம்.
தொலைபேசி இல :- 081-2396510

பெயர் :- Ms. H. M. S. P. ஹேரத்,
முகவரி :- ஒட்டுண்ணியியல் திணைக்களம், வைத்திய பீடம்,
பேராதனைப் பல்கலைக்கழகம்.
தொலைபேசி இல :- 071-7555495

Part II

அனுமதிப் பத்திரம் :-

நான் மேலே குறிப்பிடப்பட்ட தகவல்களை சுயமாக வாசித்து கிரகித்துக் கொண்டேன். சிலபுரியாத விடயங்களை, அது தொடர்பாக கேள்வி கேட்டு அறிந்து கொள்வேன். நான் எனது முழு மனதுடன் இப்பரிசோதனையில் கலந்து கொள்வதற்கான சம்மதத்தை தெரிவிக்கின்றேன்.

பங்கு கொள்ளும் இல(Serial No) :- ……………………………………..

பங்கு கொள்பவரின் பெறு விரல் அடையாளம்(Finger Print) :-
…………………………

திகதி :- ………………………………..

Questionnaire

Questionnaire for *Trichomonas* Research

Patient Serial No:

1)	We mount	**1.**	+ ve	**2**	- ve
2)	Giemsa staining	**1.**	+ ve	**2**	- ve
3)	Culture	**1.**	+ ve	**2.**	- ve
4)	PCR 1	**1.**	+ ve	**2.**	- ve
5)	PCR 2	**1.**	+ ve	**2.**	- ve
6)	PCR 3	**1.**	+ ve	**2.**	- ve
7)	Gender	**1.**	Male	**2.**	Female
8)	Civil status:	**1.**	Single	**2.**	Married
		**3.**	Living together	**4.**	Divorced
		**5.**	Widowed		
9)	Age:	**1.**	15-25 years	**2.**	26-35 years
		**3.**	36-45 years	**4.**	46-55 years
10)	Area				

11)	Highest level of education:	**1.**	1-5 grade	**2.**	6-10 grade
		**3.**	G.C.E. O/L	**4.**	G.C.E. A/L
		**5.**	Diploma/Degree	**6.**	No schooling
12)	Occupation:	**1.**	Unemployed	**2.**	Student
		**3.**	CSW	**4.**	Retired
		**5.**	Employed as ………….		
13	Reasons for attendance:	**1.**	Voluntary	**2.**	Ref. OPD
		**3.**	Ref. GP	**4.**	Ref. Ward
		**5.**	Ref. Courts	**6.**	Ref. Blood bank
		**7.**	Contacts	**8.**	Clinic follow up
		**9.**	Medico legal	**10.**	others
14	Symptoms:	**1.**	None	**2.**	Genital discharge & odor
		**3.**	Dysuria	**4.**	Warts
		**5.**	Genital ulcer	**6.**	Ulcer
		**7.**	Pelvic pain	**9.**	Other
15)	Duration of symptoms:	**1.**	Not applicable	**2.**	1-3
	(days)	**3.**	4-7	**4.**	8-14
		**5.**	Over 14	**6.**	Unknown
16)	Medication:	**1.**	None	**2.**	Antibiotics
	(14 days)	**3.**	Other		

17	Contraception	**1**	no	**2**	IUCD
		**3**	oRAL	**4**	Condom
		**5.**	Tubal ligation	**6**	Injection
		**7.**	Natural	**8.**	other
18.	Menstrual cycle	**1**	regular	**2**	Non regular
		**3**	NA LMP		
19)	Pregnant:	**1.**	No	**2.**	Yes
		**3.**	Uncertain		
20)	Miscarriages/still birth:	**1.**	No	**2.**	Yes
21	Termination of pregnancy	**1.**	No	**2.**	Yes
22)	Sex contact:	**1.**	None	**2.**	Sri Lankan
	(12 months)	**3.**	Foreign		
23)	Type of partner:	**1.**	None	**2.**	Marital/Regular partner
	(12 months)	**3.**	Non regular partner	**4.**	Commercial partner
		**5**	2 & 3	**6.**	2 & 4
		**7.**	3 & 4		
24)	Sexual orientation:	**1.**	Heterosexual (Male)	**2.**	Bisexual
		**3.**	Homosexual		
25)	No. of partners:	**1.**	One	**2.**	Two

		**3.**	Three	**4.**	Four
		**5.**	Five or more	**6.**	None/NA
26)	Condom use at last sex:	**1.**	Yes	**2.**	No
		**3.**	None/NA		
27)	Condom use:	**1.**	Always	**2.**	Never
	(last 3 months)	**3.**	Sometimes	**4.**	NA
28)	Substance abuse:	**1.**	None/NA	**2.**	Narcotics (Inhalation/oral)
	(12 months)	**3.**	Alcohol	**4.**	IDU
29)	Previous STD:	**1.**	None	**2.**	GC
		**3.**	Syphilis	**4.**	Herpes
		**5.**	Chlamydia/NGC	**6.**	Warts
		**7.**	Other		
30)	Ever had an HIV test:	**1.**	Never	**2.**	Negative
		**3.**	Positive	**4.**	Tested but results not sure
31)	Age at first sex:	**0.**	<15 years		
		**1.**	15-25 years	**2.**	26-35 years
		**3.**	36-45 years	**4.**	46-55 years
32)	Total No. of partners:	**1.**	1	**2.**	2
	(last 3 months)	**3.**	3-5	**4**	6-10
		**5**	11-20	**6**	>21
		**7**	>50	**8**	>100

33)	Total No. of partners:	**1.**	1	**2.**	2
	(last 1 year)	**3.**	3-5	**4**	6-10
		**5**	11-20	**6**	>21
		**7**	>50	**8**	>100
		**9**	>1000		
34)	Total No. of partners:	**1.**	1	**2.**	2
	(life time)	**3.**	3-5	**4**	6-10
		**5**	11-20	**6**	>21
		**7**	>50	**8**	>100
		**9**	>1000	**10**	>10000
35)	Signs:	**1.**	None	**2.**	Genital discharge
		**3.**	Inguinal LN	**4.**	Genital warts
		**5.**	Genital ulcer	**6.**	Rash
		**7.**	Pelvic tenderness	**8.**	Other
36	Urethral smear	1	Not done	2	iugnd
		3	<5 pus cells/NAD	4	5-9 pus cells/NAD
		5	>10 pus cells/NAD	6	OTHER
37	Urethral GC culture	1	Not done	2	-ve
		3	+ ve	4	NA
38	Urethral chlamedia	1	Not done	2	-ve
		3	+ ve	4	intermediate
		5	NA		

39	Vaginal smear	1	Not done	2	-ve
		3	IUGND	4	CANDIDA
		5	TV	6	Clue cells
		7	lactobacilli	8	6 & 7
		9	other		
40	Pap smear	1	Not done	2	UNSATISFACTORY
		3	NILM		
41	Hsv Ag ELISA	1	Not done	2	-ve
		3	+ ve	4	NA
42	VDRL	1	Not done	2	Non reactive
		3	Prev. reactive	4	reactive
43	TPPA	1	Not done	2	Non reactive
		3	Prev. reactive	4	reactive
44	HIV SCREENING TEST	1	Not done	2	-ve
		3	PREV + ve	4	+ VE
45	HIV CONFIRMATORY TEST	1	Not done	2	-ve
		3	PREV + ve	4	+ VE
46.	HEP Bs Ag	1	Not done	2	-ve
		3	PREV + ve	4	+ VE

27) Provisional diagnosis:

1	No STD related Illness	2	BV
3	HSV	4	Syphilis
5	NGU	6	Genital Warts
7	HIV	8	PID
9	Candiditis	10	Chlamydial Infection
11	Trichomoniasis	12	Fungal infections
13.	Other		

28. Remarks
