# Supplementary material for: Comparison of diagnostic methods and analysis of socio-demographic factors associated with Trichomonas vaginalis infection in Sri Lanka
Source: PLoS One. 2021 Oct 13;16(10):e0258556. doi: 10.1371/journal.pone.0258556 (PMC8513885; doi:10.1371/journal.pone.0258556)
Supplement: S3 Appendix — Comparison of the wet mount, Giemsa staining, culture, PCR using TFR1/2, TV16Sf/r, and TVK3/7 and expanded gold standard. (DOCX) [file pone.0258556.s006.docx]

**3.2.5 Calculation of sensitivity and specificity of diagnostic tests**

Sensitivity and specificity of each diagnostic tests were calculated compared to the expanderd gold standard defined by using the following formula (Crucitti et al., 2003). A specimen was considered as true positive for *T. vaginalis,* if it was positive by genus specific primer set and one out of two primer sets (combined results).

| **Test results** |  | **Infection** | |  |
| --- | --- | --- | --- | --- |
|  |  | **Positive** | **Negative** |  |
|  | **Positive** | True Positive | False Positive | Total test positive |
|  | **Negative** | False Negative | True Negative | Total test Negative |
|  |  | Total number with infection | Total number without infection | Total |

$$Sensitivity=\frac{True positive}{True positive+True negative} \times100$$

$$Specificity=\frac{True negative}{True negative+False positive} \times100$$

$$Positive predictive value (PPV)=\frac{True positive}{Total test positive} \times100$$

$$Negative predictive value (NPV)=\frac{True negative}{Total test negative} \times100$$

Comparison of each diagnostic test with expanded gold standard is shown in Table 1.

**Table 1 Comparison of wet mount microscopy and expanded gold standard**

| Wet mount microscopy | Expanded gold standard | | Total |
| --- | --- | --- | --- |
|  | Positive | Negative |  |
| Positive | 6 | 0 | 6 |
| Negative | 11 | 368 | 379 |
| Total | 17 | 368 | 385 |

Sensitive of wet mount microscopy = $\frac{6}{6+11} \times100$ = 35.30%

Specificity of wet mount microscopy = $\frac{368}{368+0} \times100$ = 100.00%

PPV of wet mount microscopy = $\frac{6}{6} \times100$ = 100.00%

NPV of wet mount microscopy = $\frac{368}{379} \times100$ = 97.10%

**Table 2 Comparison of Giemsa staining and expanded gold standard**

| Giemsa staining | Expanded gold standard | | Total |
| --- | --- | --- | --- |
|  | Positive | Negative |  |
| Positive | 6 | 0 | 6 |
| Negative | 11 | 368 | 379 |
| Total | 17 | 368 | 385 |

Sensitive of Giemsa staining microscopy = $\frac{6}{6+11} \times100$ = 35.30%

Specificity of Giemsa staining microscopy = $\frac{368}{368+0} \times100$ = 100.00%

PPV of wet mount microscopy = $\frac{6}{6} \times100$ = 100.00%

NPV of wet mount microscopy = $\frac{368}{379} \times100$ = 97.10%

**Table 3 Comparison of culture and expanded gold standard**

| **Culture** | Expanded gold standard | | Total |
| --- | --- | --- | --- |
|  | Positive | Negative |  |
| Positive | 7 | 0 | 7 |
| Negative | 10 | 368 | 378 |
| Total | 17 | 368 | 385 |

Sensitive of culture= $\frac{7}{6+11} \times100$ = 41.20%

Specificity of culture = $\frac{368}{368+0} \times100$ = 100.00%

PPV of wet mount microscopy = $\frac{7}{7} \times100$ = 100.00%

NPV of wet mount microscopy = $\frac{368}{378} \times100$ = 97.35%

**Table 4 Comparison of PCR using** TFR1/2 **primer set and expanded gold standard**

| PCR using TFR1/2 primer set | Expanded gold standard | | Total |
| --- | --- | --- | --- |
|  | Positive | Negative |  |
| Positive | 17 | 0 | 17 |
| Negative | 0 | 368 | 368 |
| Total | 17 | 368 | 385 |

Sensitive of PCR using TFR1/2= $\frac{17}{0+17} \times100$ = 100.00%

Specificity of PCR using TFR1/2= $\frac{368}{368+0} \times100$ = 100.00%

PPV of wet mount microscopy = $\frac{17}{17} \times100$ = 100.00%

NPV of wet mount microscopy = $\frac{368}{368} \times100$ = 100.00%

**Table 5 Comparison of PCR using** TV16Sf/r **primer set and expanded gold standard**

| PCR using TV16Sf/r primer set | Expanded gold standard | | Total |
| --- | --- | --- | --- |
|  | Positive | Negative |  |
| Positive | 15 | 0 | 15 |
| Negative | 2 | 368 | 370 |
| Total | 17 | 368 | 385 |

Sensitive of PCR using TV16Sf/r= $\frac{15}{2+15} \times100$ = 88.20%

Specificity of PCR using TV16Sf/r = $\frac{368}{368+0} \times100$ = 100.00%

PPV of wet mount microscopy = $\frac{15}{15} \times100$ = 100.00%

NPV of wet mount microscopy = $\frac{368}{370} \times100$ = 99.46%

**Table 6 Comparison of PCR using** TVK3/7 **primer set and expanded gold standard**

| PCR using TVK3/7 primer set | Expanded gold standard | | Total |
| --- | --- | --- | --- |
|  | Positive | Negative |  |
| Positive | 13 | 0 | 13 |
| Negative | 4 | 368 | 372 |
| Total | 17 | 368 | 385 |

Sensitive of PCR using TVK3/7= $\frac{13}{4+13} \times100$ = 76.50%

Specificity of PCR using TVK3/7= $\frac{368}{368+0} \times100$ = 100.00%

PPV of wet mount microscopy = $\frac{13}{13} \times100$ = 100.00%

NPV of wet mount microscopy = $\frac{368}{372} \times100$ = 98.92%

Sensitivity and specificity of each diagnostic test were tabulated in table 7.

**Table 7 Sensitivity and specificity of each diagnostic test**

| **Diagnostic test** | **Sensitivity** | **specificity** | **PPV (%)** | **NPV (%)** |
| --- | --- | --- | --- | --- |
| Wet mount microscopy | 35.30% | 100% | 100.00 | 97.10 |
| Giemsa staining | 35.30% | 100% | 100.00 | 97.10 |
| Culture | 41.20% | 100% | 100.00 | 97.35 |
| PCR |  |  |  |  |
| TFR1/2 Primer set | 100% | 100% | 100.00 | 100.00 |
| TV16Sf/r primer set | 88.2% | 99.46% | 100.00 | 99.46 |
| TVK3/7 primer set | 76.5% | 98.4% | 100.00 | 98.92 |
